# SARS-CoV-2 Antibody Prevalence and Population-Based Death Rates, Greater Omdurman, Sudan

**DOI:** 10.3201/eid2805.211951

**Published:** 2022-05

**Authors:** Wendelin Moser, Mohammed Ahmed Hassan Fahal, Elamin Abualas, Shahinaz Bedri, Mahgoub Taj Elsir, Mona Fateh El Rahman Omer Mohamed, Abdelhalim Babiker Mahmoud, Amna Ismail Ibrahim Ahmad, Mohammed A. Adam, Sami Altalib, Ola Adil DafaAllah, Salahaldin Abdallah Hmed, Andrew S. Azman, Iza Ciglenecki, Etienne Gignoux, Alan González, Christine Mwongera, Manuel Albela Miranda

**Affiliations:** Médecins Sans Frontières, Geneva, Switzerland (W. Moser, A.S. Azman, I. Ciglenecki, A. González, C. Mwongera, M. Albela Miranda);; Médecins Sans Frontières, Khartoum (M.T. Elsir, A.B. Mahmoud, A.I.I. Ahmad, M.A. Adam, S. Altalib, O.A. DafaAllah, S.A. Hmed);; Khartoum State Ministry of Health, Khartoum, Sudan (M.AH. Fahal, E. Abualas, S. Bedri, M.T. Elsir, M.F.E.R.O. Mohamed, O.A. DafaAllah);; National Public Health Laboratory, Khartoum (E. Abualas, S. Bedri, O.A. DafaAllah);; University of Khartoum, Khartoum (A.B. Mahmoud, M.A. Adam, S. Altalib);; Johns Hopkins Bloomberg School of Public Health, Baltimore, Maryland, USA (A.S. Azman);; University of Geneva, Geneva (A.S. Azman);; Epicentre, Paris, France (E. Gignoux)

**Keywords:** COVID-19, SARS-CoV-2, severe acute respiratory syndrome coronavirus 2, viruses, respiratory infections, zoonoses, seroprevalence, antibody, excess mortality, Omdurman, Sudan, *Suggested citation for this article*: Moser W, Fahal MAH, Abualas E, Bedri S, Elsir MT, Mohamed MFERO, et al. SARS-CoV-2 antibody prevalence and population-based death rates, greater Omdurman, Sudan. Emerg Infect Dis. 2022 May [*date cited*]. https://doi.org/10.3201/eid2805.211951

## Abstract

In a cross-sectional survey in Omdurman, Sudan, during March–April 2021, we estimated that 54.6% of the population had detectable severe acute respiratory syndrome coronavirus 2 antibodies. Overall population death rates among those >50 years of age increased 74% over the first coronavirus disease pandemic year.

Many key epidemiologic and serologic characteristics of severe acute respiratory syndrome coronavirus 2 (SARS-CoV-2) remain unknown. Few seroprevalence studies have been conducted in Africa to better understand the landscape of humoral immunity. In Sudan, 32,846 confirmed cases of coronavirus disease (COVID-19) were recorded during March 13, 2020– April 10, 2021; of those, 72% were registered in the state of Khartoum alone (*1*). A study of a convenience sample of >1,000 participants from 22 neighborhoods of the city of Khartoum in March–July 2020 found that 35% of participants were positive by real time RT-PCR for SARS-CoV-2, and 18% had SARS-CoV-2 antibodies (*2*). Similar discrepancies between clinical confirmed cases and infection rates assessed by serology or PCR testing independent of symptoms have been described elsewhere in Africa (*3*–*5*).

The National Health Review Ethics Committee (no. 3-1-21), Médecins Sans Frontières Ethics Review Board (ID 2089c), and Khartoum State Ministry of Health approved this study. Before field data collection began, we visited the leader of the resistance committee for each block to obtain verbal consent. For the mortality survey, we obtained verbal consent from the head of the household. For the seroprevalence survey, we obtained written informed consent from adults and, for participants <18 years of age, first written informed consent from parents or legal guardians and second, oral assent from the participants themselves.

## The Study

Sudan’s capital, Khartoum, is a tripartite metropolis comprising Khartoum, Bahri, and Omdurman; it has >8 million inhabitants (*6*). We chose Omdurman, the largest of the 3 cities, as the study site for 2 surveys conducted in March–July 2020 (Appendix). One, a retrospective mortality survey, was conducted using a 2-stage cluster sampling methodology based on random geopoints with 2 recall periods, the prepandemic (January 1, 2019–February 29, 2020) and the pandemic period (March 1, 2020–date of survey); an adult representative of the household answered a standardized questionnaire. The second was a nested SARS-CoV-2 antibody prevalence survey; all the members of a subset of the household, regardless of age, were invited to participate in the seroprevalence study.

Capillary blood was collected on dried blood spot cards and directly tested with the STANDARD Q COVID-19 IgM/IgG Combo rapid diagnostic test (RDT) (SD–Biosensor, https://www.sdbiosensor.com). All participants who tested positive for any isotype were considered seropositive. Dried blood spot cards (Euroimmun, https://www.euroimmun.com) were transferred to the National Public Health Laboratory (NPHL; Khartoum, Sudan) for further analysis by ELISA (Anti–SARS-CoV-2 ELISA [IgG, S1 domain]; Euroimmun) to compare with the rapid test results (*7*,*8*). To adjust our seroprevalence estimates using published validation data for both ELISA and RDT tests, we conducted a meta-analysis with random effects and a Bayesian latent class model (Appendix).

**Figure 1:**
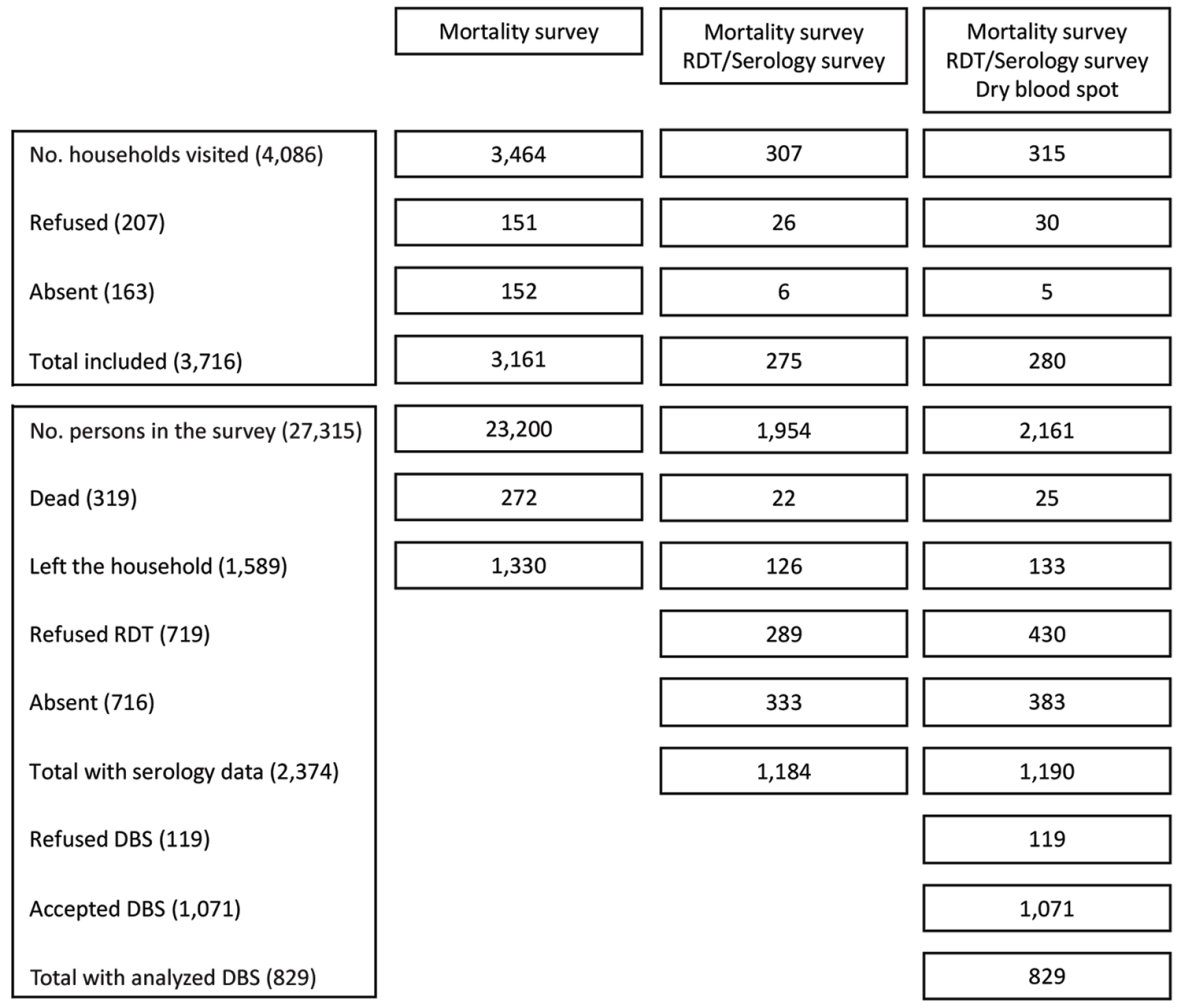
Survey flow for cross-sectional study of SARS-CoV-2 prevalence and population-based death rates, Omdurman, Sudan, 2021. DBS, dry blood spot; RDT, rapid diagnostic test.

During March 1–April 10, 2021, a total of 2,374 (62.3%) participants from 555 households (Figure 1) agreed to provide blood; 34.3% (95% CI 32.4%–36.2%; Table 1) of them had detectable SARS-CoV-2 antibodies (IgM, IgG, or both). After adjusting for immunoassay performance for detecting previous infections, we estimated a seroprevalence of 54.6% (95% CI 51.4%–57.8%), noting a clear increase of seroprevalence risk with age (Table 1). We found the highest seroprevalence of 80.7% (95% CI 71.7%–89.7%) among participants >50 years of age. Assuming a population size of 3,040,604 for Omdurman on the basis of the data collected in the survey and the data provided by the Ministry of Planning, we estimate that 1,660,170 (95% CI 1,458,225–1,863,936) persons had been infected by SARS-CoV-2 at the time of the survey.

**Table 1:** SARS-CoV-2 antibody seroprevalence test results by age group in cross-sectional survey, Omdurman, Sudan*

Age group	RDT results				Adjusted results		
	% Positive (95% CI)	Relative risk (95% CI)	p value†		Seroprevalence (95% CI)	Relative risk (95% CI)	p value†
<5 y, = 299	18.7 (14.7–23.5)	0.4 (0.3–0.5)	<0.001		29.0 (22.4–36.9)	0.3 (0.3–0.4)	<0.001
5–19 y, = 786	30.6 (27.5–33.9)	0.6 (0.5–0.7)	<0.001		48.5 (43.3–53.9)	0.6 (0.5–0.6)	<0.001
20–34 y, = 629	35.5 (31.8–39.3)	0.7 (0.6–0.8)	<0.001		56.5 (50.5–62.8)	0.7 (0.6–0.7)	<0.001
35–49 y, = 342	39.5 (34.4–44.7)	0.8 (0.7–0.9)	0.006		63.1 (54.8–71.8)	0.8 (0.7–0.9)	<0.001
>50 y, = 319	50.2 (44.7–55.6)	Referent			80.7 (71.7–89.7)	Referent	
Overall, = 2,375	34.3 (32.4–36.2)				54.6 (51.4–57.8)		

*RDT, rapid diagnostic test; SARS-CoV-2, severe acute respiratory syndrome coronavirus 2. †p values indicate the difference in relative risk between the oldest age group (≥50 y) as reference and the other age groups.

**Figure 2:**
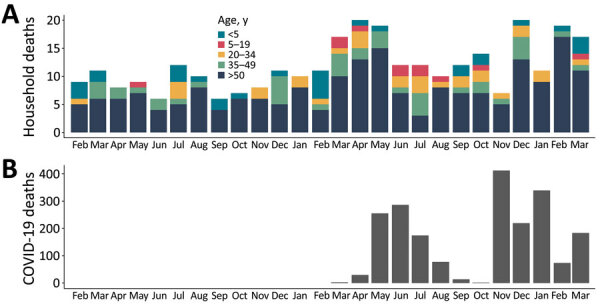
Comparison of estimated and reported deaths from coronavirus disease, Sudan, January 2019–April 2021. A) Distribution of all deaths as reported in a population-based cross-sectional survey in the city of Omdurman, Sudan. B) Official registered COVID-19–related deaths across Sudan.

We found evidence of significant clustering of seropositivity within households; 364 households (65.6%) had >1 positive household member. Living with a person who was seropositive led to a 1.68-fold (odds ratio [OR] 95% CI 1.35–2.08; p<0.001) increase in the odds of being seropositive (Appendix). Among the 4,086 households visited (Figure 1), we enumerated 27,315 persons who had been a household member at some time after January 1, 2019. Among them, 319 deaths were reported, including 206 (64.6%) among persons >50 years of age and 30 (9.4%) among children <5 years of age. The deaths increased in 2020 during the pandemic period, consistent with the reported countrywide confirmed COVID-19 deaths (Figure 2).

The overall death rate for the whole recall period was 0.16 (95% CI 0.13–0.18) deaths/10,000 population/day (Table 2). The crude death rate significantly increased by 67% (95% CI 32%–110%) from 0.12 (0.10–0.14) deaths/10,000 population/day for the prepandemic period to 0.20 (0.16–0.23) deaths/10,000 population/day for the pandemic period. This difference was even more pronounced among those *>*50 years of age; deaths increased 74% (95% CI 30%–133%; p<0.001) between the 2 periods. (Table 2). On the basis of on our estimates of the population size of Omdurman and the death rates, we estimated 7,113 excess deaths (95% CI 5,015–9,505) during the pandemic period and that 5,125 (95% CI 4,165–6,226) of these occurred in persons *>*50 years of age.

**Table 2:** Reported death rates for the prepandemic and pandemic periods from cross-sectional SARS-CoV-2 survey, Omdurman, Sudan*

Age group	Overall			Prepandemic period			Pandemic period			Rate ratio	
	No. deaths	Rate (95% CI)		No. deaths	Rate (95% CI)		No. deaths	Rate (95% CI)		Rate ratio (95% CI)	p value
<5 y	30	0.19 (0.10–0.28)		18	0.22 (0.11–0.32)		12	0.17 (0.04–0.30)		0.77 (0.34–1.70)	0.613
5–19 y	13	0.02 (0.01–0.03)		2	0.00 (0.00–0.01)		11	0.03 (0.01–0.05)		Referent	NA
20–34 y	30	0.05 (0.03–0.07)		10	0.04 (0.01–0.06)		20	0.07 (0.04–0.11)		1.75 (0.78–4.19)	0.199
35–49 y	40	0.12 (0.09–0.16)		16	0.09 (0.05–0.14)		24	0.15 (0.09–0.21)		1.67 (0.85–3.36)	0.149
>50 y	206	0.78 (0.65–0.91)		80	0.57 (0.45–0.69)		126	0.99 (0.79–1.20)		1.74 (1.30–2.33)	<0.001
Total	319	0.16 (0.13–0.18)		126	0.12 (0.10–0.14)		193	0.20 (0.16–0.23)		1.67 (1.32–2.10)	<0.001

*No. deaths per category are reported rates. NA, not applicable; SARS-CoV-2, severe acute respiratory syndrome coronavirus 2.

## Conclusions

Our findings indicate that mortality rates in the overall population of Omdurman increased by 67% during the first pandemic year; the highest increase (74%) was among the population *>*50 years of age. We estimated an excess of 7,113 all-cause deaths during the pandemic period, compared with 287 COVID-19–related deaths officially reported for Omdurman; these data were obtained from the Khartoum Ministry of Health. We have considered the potential limitation of having a recall period >2 years for mortality estimates, which could introduce bias for deaths occurring at the beginning of the recall period. Surveyors were trained to be aware of this factor to mitigate those bias (Appendix).

The crude seroprevalence estimate shows how widespread SARS-CoV-2 infection was, affecting all age groups, especially persons *>*50 years of age. However, the estimates based on RDT results might have underestimated the seroprevalence as a result of several limitations. First, we conducted our survey 1 year after the earliest SARS-CoV-2 infection was detected in Sudan, so a varying degree of antibody decay over time could be expected (*9*,*10*). Second, when antibodies remain present in the blood, their detection is limited by the performance of the RDT (*11*). To overcome those limitations, we adjusted the crude results; we observed a 20% increase in the overall seroprevalence. With that estimation we calculated that the number of infections was 50 times higher than the number of COVID-19 cases recorded by the end of the survey, which was consistent with other case-to-infection ratios in low-income settings in Africa and Asia (*12*,*13*). Despite this high seroprevalence, another wave of infection occurred right after the survey (May–June 2021); comparing it with the previous wave, we saw that fewer cases but more deaths per case were reported. Three more waves occurred during September 2021–January 2022, the latest one reporting a record number of weekly cases (*14*). No sequencing data was available as of January 2022; therefore, it was impossible to discuss the emergence of new variants and their impact on the new waves of infections given the prior seroprevalence we estimated in this survey.

In summary, this population-based cross-sectional survey in Omdurman, Sudan, demonstrated significantly higher death rates during the COVID-19 pandemic compared with those of the prepandemic period, particularly affecting persons *>*50 years of age. We also found elevated SARS-CoV-2 seropositivity, affecting older populations the most. Our results suggest that Omdurman, one of the largest population centers in Africa, was severely affected by the COVID-19 pandemic and that excess mortality rates were much higher than reported COVID-19 deaths.

Appendix Additional information about severe acute respiratory syndrome coronavirus 2 seroprevalance and death rates in Sudan.

## References

[1] Mukhtar MM, Khogali M. The accelerating COVID-19 epidemic in Sudan. Nat Immunol. 2021;22:797–8.<jrn[REMOVED IF= FIELD]><jrn[REMOVED IF= FIELD]></jrn></jrn> 10.1038/s41590-021-00950-0 34035525

[2] TEPHINET. Sudan FETP conducts targeted testing for COVID-19 in Khartoum state. 2020 [cited 2021 Feb 2]. https://www.tephinet.org/sudan-fetp-conducts-targeted-testing-for-covid-19-in-khartoum-state<eref[REMOVED IF= FIELD]><eref[REMOVED IF= FIELD]></eref></eref>

[3] Musa HH, Musa TH, Musa IH, Musa IH, Ranciaro A, Campbell MC. Addressing Africa’s pandemic puzzle: Perspectives on COVID-19 transmission and mortality in sub-Saharan Africa. Int J Infect Dis. 2021;102:483–8.<jrn[REMOVED IF= FIELD]><jrn[REMOVED IF= FIELD]></jrn></jrn> 10.1016/j.ijid.2020.09.1456 33010461 PMC7526606

[4] Lawal Y. Africa’s low COVID-19 mortality rate: A paradox? Int J Infect Dis. 2021;102:118–22.<jrn[REMOVED IF= FIELD]><jrn[REMOVED IF= FIELD]></jrn></jrn> 10.1016/j.ijid.2020.10.038 33075535 PMC7566670

[5] Oladipo EK, Ajayi AF, Odeyemi AN, Akindiya OE, Adebayo ET, Oguntomi AS, et al. Laboratory diagnosis of COVID-19 in Africa: availability, challenges and implications. Drug Discov Ther. 2020;14:153–60.<jrn[REMOVED IF= FIELD]><jrn[REMOVED IF= FIELD]></jrn></jrn> 10.5582/ddt.2020.03067 32908070

[6] Imperial College London. Report 39—characterizing COVID-19 epidemic dynamics and mortality under-ascertainment in Khartoum, Sudan. 2021 [cited 2021 Feb 2]. http://www.imperial.ac.uk/medicine/departments/school-public-health/infectious-disease-epidemiology/mrc-global-infectious-disease-analysis/covid-19/report-39-sudan<eref[REMOVED IF= FIELD]><eref[REMOVED IF= FIELD]></eref></eref>

[7] Higgins RL, Rawlings SA, Case J, Lee FY, Chan CW, Barrick B, et al. Longitudinal SARS-CoV-2 antibody study using the Easy Check COVID-19 IgM/IgG™ lateral flow assay. PLoS One. 2021;16:e0247797.<jrn[REMOVED IF= FIELD]><jrn[REMOVED IF= FIELD]></jrn></jrn> 10.1371/journal.pone.0247797 33661960 PMC7932143

[8] Pavlova IP, Nair SS, Kyprianou N, Tewari AK. The rapid coronavirus antibody test: can we improve accuracy? Front Med (Lausanne). 2020;7:569.<jrn[REMOVED IF= FIELD]> <jrn[REMOVED IF= FIELD]></jrn></jrn> 10.3389/fmed.2020.00569 32984390 PMC7492556

[9] Wang H, Yuan Y, Xiao M, Chen L, Zhao Y, Haiwei Zhang, et al. Dynamics of the SARS-CoV-2 antibody response up to 10 months after infection. Cell Mol Immunol. 2021;18:1832–4.<jrn[REMOVED IF= FIELD]><jrn[REMOVED IF= FIELD]></jrn></jrn> 10.1038/s41423-021-00708-6 34099890 PMC8182358

[10] Chia WN, Zhu F, Ong SWX, Young BE, Fong S-W, Le Bert N, et al. Dynamics of SARS-CoV-2 neutralising antibody responses and duration of immunity: a longitudinal study. Lancet Microbe. 2021;2:e240–9.<jrn[REMOVED IF= FIELD]><jrn[REMOVED IF= FIELD]></jrn></jrn> 10.1016/S2666-5247(21)00025-2 33778792 PMC7987301

[11] Uwamino Y, Wakui M, Aoki W, Kurafuji T, Yanagita E, Morita M, et al.; Keio Donner Project Team. Evaluation of the usability of various rapid antibody tests in the diagnostic application for COVID-19. Ann Clin Biochem. 2021;58:174–80.<jrn[REMOVED IF= FIELD]><jrn[REMOVED IF= FIELD]></jrn></jrn> 10.1177/0004563220984827 33334135 PMC7797350

[12] Wiens KE, Mawien PN, Rumunu J, Slater D, Jones FK, Moheed S, et al. Seroprevalence of severe acute respiratory syndrome coronavirus 2 IgG in Juba, South Sudan, 2020. Emerg Infect Dis. 2021;27:1598–606.<jrn[REMOVED IF= FIELD]> <jrn[REMOVED IF= FIELD]></jrn></jrn> 10.3201/eid2706.210568 34013872 PMC8153877

[13] Bhuiyan TR, Hulse JD, Hegde ST, Akhtar M, Islam T, Khan ZH, et al. SARS-CoV-2 seroprevalence before Delta variant surge, Chattogram, Bangladesh, March–June 2021. Emerg Infect Dis. 2022;28:429–31.<unknown[REMOVED IF= FIELD]><unknown[REMOVED IF= FIELD]></unknown></unknown> 10.3201/eid2802.211689 35076007 PMC8798688

[14] World Health Organization. Health emergency dashboard: Sudan.2022 [cited 2022 Jan 19]. https://covid19.who.int/region/emro/country/sd<eref[REMOVED IF= FIELD]><eref[REMOVED IF= FIELD]></eref></eref>

