# Supplementary material for: SARS-CoV-2 Antibody Prevalence and Population-Based Death Rates, Greater Omdurman, Sudan
Source: Emerg Infect Dis. 2022 May;28(5):1026–30. doi: 10.3201/eid2805.211951 (PMC9045432; doi:10.3201/eid2805.211951)
Supplement: Appendix — Additional information about severe acute respiratory syndrome coronavirus 2 seroprevalance and death rates in Sudan. [file 21-1951-Techapp-s1.pdf]

Article DOI: https://doi.org/10.3201/eid28052.11951

SARS-CoV-2 Antibody Prevalence and Population-Based Death Rates, Greater Omdurman, Sudan

Appendix

Methodology

Study Area and Design

Sudan's capital Khartoum is a tripartite metropolis comprising Khartoum, Bahri and Omdurman with a total of 8 million inhabitants (*1*), located at the confluence of the White and Blue Nile. Omdruman, the largest among the three cities was chosen as study site, which included 2 surveys: i) a retrospective mortality survey using a 2–stage cluster sampling methodology based on random geopoints and ii) a nested SARS-CoV-2 antibody prevalence survey. For the mortality survey the recall period was divided into 2 periods: the prepandemic (January 1, 2019–February 29, 2020) and the pandemic (March 1, 2020–day of the survey) periods.

The primary objective of the survey was to estimate death rate for persons ≥50 years and the overall seroprevalence of SARS-CoV-2 antibodies in Omdurman. The secondary objectives included age group–specific (<5, 5–19, 20–34, 35–49, ≥50 years) seroprevalences, risk factors for seropositivity, health seeking behavior, and access to healthcare among the people living in greater Omdurman. Additionally, the sensitivity and specificity of the rapid serologic test (RDT) was compared to that of the enzyme–linked immunosorbent assay (ELISA).

Procedures

The Ministry of Planning provided a point file containing each middle point of polygons representing a residential parcel, which was considered as a household for this survey. Proportional to the total number of parcels in the 34 administrative units in greater Omdurman (Appendix Figure 1), 140 points were randomly chosen using the random generator software of ArcGIS version 10.5 (https://www.arcgis.com), identifying the first household of a cluster with a

total of 30 households. The remaining 29 households were chosen in closest proximity to the first household. All households were included in the mortality survey, whereas the seroprevalence survey included 4 randomly selected households per cluster, of which all family members without any age restriction were invited to participate. Participants were excluded if they were absent after 3 attempted visits. Additionally, dried blood spots (DBS) were collected from 2 out of the 4 households participating in the seroprevalence survey. Previously trained medical doctors forming the survey team carried out the RDT and DBS collection.

For the mortality survey, a questionnaire was administered to the head of each household, adapted from recent World Health Organization recommendations for identifying mortality from COVID-19 (*2*). Information gathered included demographics of all household members, details on deceased household members, comorbidities, COVID-19 testing, and health seeking behavior (Appendix Tables 1–6). For the seroprevalence survey, each participant was asked individually about past symptoms related to COVID-19, ongoing treatment, exposure to a suspect or confirmed COVID-19 case, and other risk factors (Appendix Tables 7–11).

Sample Size Calculation

The sample size calculations were performed using ENA 2020 software (https://smartmethodology.org). The sample size for the mortality survey was based on deaths of persons ≥50 years of age (0.73 deaths/10,000 persons/day) (*3*), with a precision of ±0.2, a design effect of 1.2, and a household size of 6 persons, resulting in a required 3,637 households. For the seroprevalence survey, a SARS-CoV-2 antibody prevalence of 34% in the population was assumed based on the preliminary results from a study in Khartoum (*4*). To allow for age stratification, the sample size was based on the smallest age group (≥50 years), representing ≈11.5% of the population (*4*). To obtain a precision of ±5%, a 5% type 1 error, and 5% inconclusive results, ≥363 persons per age–group were required.

To assess the diagnostic performance of the RDT, a total of 745 samples were required for analysis by ELISA (*5*,*6*). Specifically, 191 positive and 554 negative samples were needed to confirm the sensitivity (97.0%, precision ±2.5%) and specificity (96.2%, precision ±1.7%) according to manufacturer.

Ethics

Ethical approval was obtained from the National Health Review Ethics Committee (No. 3–1–21), Médecins Sans Frontières Ethics Review Board (ID 2089c) and Khartoum State Ministry of Health. In addition, the 3 localities of greater Omdurman (Omdurman, Umbedda, and Kereri) were informed and authorization received before seeking authorization from the administrative units within the localities. Before beginning field data collection, we visited the leader of the resistance committee for each block to obtain verbal consent. For the mortality survey, we obtained verbal consent from the head of the household. For the seroprevalence survey, we obtained written informed consent from adults; for participants <18 years of age, we first obtained written informed consent from parents or legal guardians and then oral assent from the participants themselves.

Diagnostics

For practical reasons and to minimize refusals, we used the least invasive method with capillary blood collection for rapid serologic testing (STANDARD Q COVID-19 IgM/IgG Combo, https://sdbiosensor.com). Indeed, within the team we discussed intensively about whether we should be reporting the estimated infection fatality rate using the results from the survey. In the end we decided not to do it because we did not have reliable mortality data for Omdurman outside of the survey; also, we could not be sure of the cause of death for the excess mortality estimated (Appendix Table 1). The general agreement was that including this estimation could have been misleading. The IFR projected for Africa range was from 0.004% in Botswana and Central African Republic to 0.67% in South Africa (*1*) or 1.53% in Nigeria (*2*). All participants either positive for IgM, positive for IgG or positive for IgM and IgG, based on the RDT were considered positive for SARS-CoV-2 antibodies. According to the manufacturer, the RDT has a sensitivity of 96.9% (95% CI 91.3–99.4) and specificity of 96.2% (93.2–98.2). The DBS cards were transferred to the National Public Health Laboratory in Khartoum for further analysis by ELISA (EUROIMMUN Anti–SARS-CoV-2 ELISA [IgG, S1 domain], Lot: E210118BQ; https://www.euroimmun.com) following standard operating procedures. According to the manufacturer, the ELISA assay has a sensitivity of 94.4% and a specificity of 99.6% for detecting previous SARS-CoV-2 antibodies.

Statistical Analysis

We analyzed the data using R (https://www.r-project.org) and Stata version 15 (StataCorp, https://www.stata.com). For the crude death rates (expressed as deaths/10,000 persons/day) a design effect was assumed to weight the differences among clusters (Appendix Tables 1–3). To compare death rates between the prepandemic and pandemic periods, the rate ratio was calculated based on a 2–sided exact rate ratio test, and Fisher's exact test was applied to proportions where appropriate. For having the most accurate estimation of the seroprevalence based on the tests used in this survey, we defined 2 different approaches. First, published performance estimates for the RDT were used for a meta-analysis with random effects model (adjustment 1). The model provided a corrected estimate of the sensitivity and specificity for adjusting the crude seroprevalence. Considering the lack of a standard test for detecting SARS-CoV-2 antibodies, the waning of antibodies, and the fixed threshold for their detection by RDT (*7*,*8*), a second adjustment (adjustment 2) was done. The survey's ELISA results were combined with the performance estimation from the previously defined random effects model for both the ELISA and RDT and used as inputs for a Bayesian latent-class model (BLCM) (*9*–*11*), resulting in a RDT performance estimation used as adjustment. For calculating the β distributions of the priors for the BLCM, the BetaBuster software (*12*) was used. When comparing the results of the RDT with ELISA only the positive for IgG or IgG/IgM were considered positive. Risk factors associated with a positive RDT were assessed with a logistic regression model. To estimate excess deaths, SARS-CoV-2 infections, and infection fatality rate, the survey results were extrapolated as our population estimation and average household size multiplied by the number of households provided by the Ministry of Planning.

Adjustment Description, Code and Outputs

Adjustment 1 – Meta-Analysis with Random Effects Model

For adjustment 1, we estimated sensitivity and specificity of the tests using a univariate random effects model following the methods described by Shim et al. (*13*) The idea behind this model is to account for the existing performance data for the tests to improve the estimation of the results. For the model, we conducted a meta-analysis of the existing published performance data for each of the tests (RDT and ELISA) using the Metaprop function from the R package Meta. For the sensitivity and specificity estimation of the RDT we gathered data from 7 studies.

Overall, 6 studies derived from the FIND (the Global Alliance for Diagnostics) database (*14*) and data were filtered by assay name (Standard Q COVID-19 IgM/IgG Combo Test), target (IgG/IgM), and all periods. The last study was from the US Food and Drug Administration (FDA) independent evaluations of COVID-19 serologic tests (*15*). For the ELISA analysis, we used 4 studies: 2 independent (*16*,*17*), 1 from the US FDA (*18*), and 1 from the FIND database (*19*). For the RDT, the resulting sensitivity was 76.6% (95% CI 67.8–83.6) and specificity 99.0% (95% CI 98.3–99.4), and for ELISA, sensitivity was 76.9% (95% CI 61.1–87.6) and specificity 99.1% (97.7–99.7). To estimate the adjusted seroprevalence we used the epi.prev function from the R package EpiR with the estimated specificity and sensitivity of the RDT obtained from the meta-analysis.

Adjustment 2 – Bayesian Latent-Class Model

We used adjustment 2 to account for the lack of a standard for the detection of SARS-CoV-2 antibodies and the use of imperfect tests. For this purposes we used a BLCM based on the Hui-Walter paradigm, which has been widely documented (*20*–*22*) as a valuable tool to estimate the performance of diagnostic tests in scenarios similar to the ones in this survey. To develop this model we used the runjags R package (*23*) and the data of the participants with ELISA and RDT test results. We divided the data into 5 groups by participant age. We used BetaBuster (*24*) software to calculate the β distributions of the sensitivity and specificity resulted from the previous meta-analysis. This information resulted in the following prior β functions and used as parameters for the BLCM: RDT sensitivity dbeta(61.22,19.40), specificity dbeta(965.86, 10.74); ELISA sensitivity dbeta(21.77, 7.24), specificity dbeta(332.21, 4.01). On the basis of this model we estimated a RDT sensitivity and specificity of 61.9% (56.8–66.9) and 98.9% (98.2–99.5). To estimate the adjusted seroprevalence we used the epi.prev function with the dataset of all the participants in the serology part of the survey and the estimated specificity and sensitivity of the RDT from the BLCM.

RDT

```
## Studies 1-6 <- https://www.finddx.org/sarscov2-eval-antibody/
## Study 7 <- https://open.fda.gov/apis/device/covid19serology/
https://www.accessdata.fda.gov/cdrh_docs/presentations/maf/maf3274-a001.pdf
S1sens <- 0.667
S1spec <- 1
S1pos <- 96
S1neg <- 102
```

```
TP1 <- round(S1sens*S1pos)
TN1 <- round(S1spec*S1neg)
FN1 <- S1pos-TP1
FP1 <- S1neg-TN1
S2sens <- 0.719
S2spec <- 0.991
S2pos <- 166
S2neg <- 196
TP2 <- round(S2sens*S2pos)
TN2 <- round(S2spec*S2neg)
FN2 <- S2pos-TP2
FP2 <- S2neg-TN2
S3sens <- 0.577
S3spec <- 0.976
S3pos <- 317
S3neg <- 125
TP3 <- round(S3sens*S3pos)
TN3 <- round(S3spec*S3neg)
FN3 <- S3pos-TP3
FP3 <- S3neg-TN3
S4sens <- 0.820
S4spec <- 0.990
S4pos <- 579
S4neg <- 423
TP4 <- round(S4sens*S4pos)
TN4 <- round(S4spec*S4neg)
FN4 <- S4pos-TP4
FP4 <- S4neg-TN4
S5sens <- 0.807
S5spec <- 0.996
S5pos <- 262
S5neg <- 298
TP5 <- round(S5sens*S5pos)
TN5 <- round(S5spec*S5neg)
FN5 <- S5pos-TP5
FP5 <- S5neg-TN5
S6sens <- 0.897
S6spec <- 0.984
S6pos <- 483
S6neg <- 321
TP6 <- round(S6sens*S6pos)
TN6 <- round(S6spec*S6neg)
FN6 <- S6pos-TP6
FP6 <- S6neg-TN6
S7sens <- 0.763
S7spec <- 0.988
```

```
S7pos <- 30
S7neg <- 80
TP7 <- round(S7sens*S7pos)
TN7 <- round(S7spec*S7neg)
FN7 <- S7pos-TP7
FP7 <- S7neg-TN7
AuditC5 <- data.frame(TP = c(TP1, TP2, TP3, TP4, TP5, TP6, TP7),
FN = c(FN1, FN2, FN3, FN4, FN5, FN6, FN7),
FP = c(FP1, FP2, FP3, FP4, FP5, FP6, FP7),
TN = c(TN1, TN2, TN3, TN4, TN5, TN6, TN7))
AuditC5$names <- c("Study 1", "Study 2", "Study 3","Study 4", "Study 5", "Study 6", "Study
7")
sens_logit_rdt <- metaprop(AuditC5$TP, AuditC5$TP + AuditC5$FN, comb.fixed=F,
comb.random=T, sm="PLOGIT", method = "GLMM", method.ci="CP",
studlab=AuditC5$names)
spec_logit_rdt <- metaprop(AuditC5$TN, AuditC5$TN + AuditC5$FP, comb.fixed=FALSE,
comb.random=TRUE, sm="PLOGIT", method.ci="CP", studlab=AuditC5$names)
```

Outputs (Appendix Figures 1, 2)

```
##Estimation of the adjusted seroprevalence with a given sensitivity and specificity of a test
adj_serop_overall <- epi.prev(nrow(sero[test_result_cat=="Positive", ]),
nrow(sero[!is.na(test_result_cat) & outcome == "done", ]),
se = 0.766, sp = 0.990)
adj_serop_o50 <- epi.prev(nrow(sero[age_group == "[50,Inf)" & test_result_cat=="Positive", ]),
nrow(sero[age_group == "[50,Inf)" & !is.na(test_result_cat) & outcome == "done", ]),
se = 0.766, sp = 0.990)
adj_serop_35_50 <- epi.prev(nrow(sero[age_group == "[35,50)" & test_result_cat=="Positive",
]),
nrow(sero[age_group == "[35,50)" & !is.na(test_result_cat) & outcome == "done", ]),
se = 0.766, sp = 0.990)
adj_serop_20_35 <- epi.prev(nrow(sero[age_group == "[20,35)" & test_result_cat=="Positive",
]),
nrow(sero[age_group == "[20,35)" & !is.na(test_result_cat) & outcome == "done", ]),
se = 0.766, sp = 0.990)
adj_serop_5_20 <- epi.prev(nrow(sero[age_group == "[5,20)" & test_result_cat=="Positive", ]),
nrow(sero[age_group == "[5,20)" & !is.na(test_result_cat) & outcome == "done", ]),
se = 0.766, sp = 0.990)
adj_serop_0_5 <- epi.prev(nrow(sero[age_group == "[0,5)" & test_result_cat=="Positive", ]),
nrow(sero[age_group == "[0,5)" & !is.na(test_result_cat) & outcome == "done", ]),
se = 0.766, sp = 0.990)
```

ELISA

```
## Calculating the sens and spec of the ELISA test through a meta-analysis
## Study 1 <- Kr?ttgen A, Cornelissen CG, Dreher M, Hornef M, Im?hl M, Kleines M.
Comparison of four new commercial serologic assays for determination of SARS-CoV-2 IgG. J
Clin Virol. 2020;128:104394. doi:10.1016/j.jcv.2020.104394
```

```
## Study 2 <- https://open.fda.gov/apis/device/covid19serology/
https://www.accessdata.fda.gov/cdrh_docs/presentations/maf/maf3246-a001.pdf
## Study 3 <- Kohmer N, Westhaus S, R?hl C, Ciesek S, Rabenau HF. Brief clinical evaluation
of six high-throughput SARS-CoV-2 IgG antibody assays. J Clin Virol. 2020;129:104480.
doi:10.1016/j.jcv.2020.104480
## Study 4 <- https://www.finddx.org/sarscov2-eval-antibody/
S1sens_el <- 0.864
S1spec_el <- 0.962
S1pos_el <- 22
S1neg_el <- 53
TP1 <- round(S1sens_el*S1pos_el)
TN1 <- round(S1spec_el*S1neg_el)
FN1 <- S1pos_el-TP1
FP1 <- S1neg_el-TN1
S2sens_el <- 0.900
S2spec_el <- 1
S2pos_el <- 30
S2neg_el <- 80
TP2 <- round(S2sens_el*S2pos_el)
TN2 <- round(S2spec_el*S2neg_el)
FN2 <- S2pos_el-TP2
FP2 <- S2neg_el-TN2
S3sens_el <- 0.711
S3spec_el <- 1
S3pos_el <- 45
S3neg_el <- 22
TP3 <- round(S3sens_el*S3pos_el)
TN3 <- round(S3spec_el*S3neg_el)
FN3 <- S3pos_el-TP3
FP3 <- S3neg_el-TN3
S4sens_el <- 0.600
S4spec_el <- 0.99
TP4 <- 55
TN4 <- 294
FN4 <- 37
FP4 <- 2
AuditC5_el <- data.frame(TP = c(TP1, TP2, TP3, TP4),
FN = c(FN1, FN2, FN3, FN4),
FP = c(FP1, FP2, FP3, FP4),
TN = c(TN1, TN2, TN3, TN4))
AuditC5_el$names <- c("Study 1", "Study2", "Study 3", "Study 4")
sens_logit_el <- metaprop(AuditC5_el$TP, AuditC5_el$TP + AuditC5_el$FN, comb.fixed=F,
comb.random=T, sm="PLOGIT", method = "GLMM", method.ci="CP",
studlab=AuditC5_el$names)
```

spec_logit_el <- metaprop(AuditC5_el$TN, AuditC5_el$TN + AuditC5_el$FP, comb.fixed=FALSE, comb.random=TRUE, sm="PLOGIT", method.ci="CP", studlab=AuditC5_el$names)

Outputs (Appendix Figures 3, 4)

Adjustment 2 – R Code

BLCM model definition:

```
model{
## Observation layer:
# Complete observations (N=825):
for(p in 1:Populations){
Tally_RR[1:4,p] ~ dmulti(prob_RR[1:4,p], N_RR[p])
prob_RR[1:4,p] <- se_prob[1:4,p] + sp_prob[1:4,p]
}
## Observation probabilities:
for(p in 1:Populations){
# Probability of observing RDT- ELISA- from a true positive::
se_prob[1,p] <- prev[p] * ((1-se[1])*(1-se[2]) +covse12)
# Probability of observing RDT- ELISA- from a true negative::
sp_prob[1,p] <- (1-prev[p]) * (sp[1]*sp[2] +covsp12)
# Probability of observing RDT+ ELISA- from a true positive::
se_prob[2,p] <- prev[p] * (se[1]*(1-se[2]) -covse12)
# Probability of observing RDT+ ELISA- from a true negative::
sp_prob[2,p] <- (1-prev[p]) * ((1-sp[1])*sp[2] -covsp12)
# Probability of observing RDT- ELISA+ from a true positive::
se_prob[3,p] <- prev[p] * ((1-se[1])*se[2] -covse12)
# Probability of observing RDT- ELISA+ from a true negative::
sp_prob[3,p] <- (1-prev[p]) * (sp[1]*(1-sp[2]) -covsp12)
# Probability of observing RDT+ ELISA+ from a true positive::
se_prob[4,p] <- prev[p] * (se[1]*se[2] +covse12)
# Probability of observing RDT+ ELISA+ from a true negative::
sp_prob[4,p] <- (1-prev[p]) * ((1-sp[1])*(1-sp[2]) +covsp12)
}
## Priors:
# Prevalence in population [0,5):
prev[1] ~ dbeta (1,1)
# Prevalence in population [5,20):
prev[2] ~ dbeta (1,1)
# Prevalence in population [20,35):
prev[3] ~ dbeta (1,1)
# Prevalence in population [35,50):
prev[4] ~ dbeta (1,1)
# Prevalence in population [50,Inf):
prev[5] ~ dbeta (1,1)
# Sensitivity of RDT test:
se[1] ~ dbeta(61.22,19.40)T(1-sp[1], )
```

```
# Specificity of RDT test:
sp[1] ~ dbeta(965.86, 10.74)
# Sensitivity of ELISA test:
se[2] ~ dbeta(21.77, 7.24)T(1-sp[2], )
# Specificity of ELISA test:
sp[2] ~ dbeta(332.21, 4.01)
# Covariance in sensitivity between RDT and ELISA tests:
# covse12 ~ dunif( (se[1]-1)*(1-se[2]) , min(se[1],se[2]) - se[1]*se[2] ) ## if the sensitivity of these tests may be correlated
covse12 <- 0 ## if the sensitivity of these tests can be assumed to be independent
# Covariance in specificity between RDT and ELISA tests:
# covsp12 ~ dunif( (sp[1]-1)*(1-sp[2]) , min(sp[1],sp[2]) - sp[1]*sp[2] ) ## if the specificity of these tests may be correlated
covsp12 <- 0 ## if the specificity of these tests can be assumed to be independent
}
#monitor# se, sp, prev, covse12, covsp12
## Inits:
inits{
"se" <- c(0.5, 0.99)
"sp" <- c(0.99, 0.75)
"prev" <- c(0.05, 0.95, 0.05, 0.95, 0.05)
# "covse12" <- 0
# "covsp12" <- 0
}
inits{
"se" <- c(0.99, 0.5)
"sp" <- c(0.75, 0.99)
"prev" <- c(0.95, 0.05, 0.95, 0.05, 0.95)
# "covse12" <- 0
# "covsp12" <- 0
}
## Data:
data{
"Populations" <- 5
"N_RR" <- c(60, 312, 214, 136, 103)
"Tally_RR" <- structure(c(44, 1, 9, 6, 161, 25, 56, 70, 126, 16, 36, 36, 68, 10, 24, 34, 44, 8, 13, 38), .Dim = c (4,5))
}
##Model call
# Set different starting values for the three different chains to assess convergence
inits1 = list(".RNG.name" ="base::Mersenne-Twister",
".RNG.seed" = 100022)
inits2 = list(".RNG.name" ="base::Mersenne-Twister",
".RNG.seed" = 300022)
inits3 = list(".RNG.name" ="base::Mersenne-Twister",
".RNG.seed" = 500022)
```

```
run.jags("./autohw.bug",
inits=list(inits1, inits2, inits3),
n.chains = 3,
burnin = 10000,
sample = 100000,
adapt = 1000,
)
## Calculating the adjusted seroprevalence
adj_bay_serop_overall <- epi.prev(nrow(sero[test_result_cat=="Positive", ]), method = "c-p",
nrow(sero[!is.na(test_result_cat) & outcome == "done", ]),
se=0.619, sp=0.989)
adj_bay_serop_o50 <- epi.prev(nrow(sero[age_group == "[50,Inf)" &
test_result_cat=="Positive", ]),
nrow(sero[age_group == "[50,Inf)" & !is.na(test_result_cat) & outcome == "done", ]),
0.619, 0.989)
adj_bay_serop_35_50 <- epi.prev(nrow(sero[age_group == "[35,50)" &
test_result_cat=="Positive", ]),
nrow(sero[age_group == "[35,50)" & !is.na(test_result_cat) & outcome == "done", ]),
0.619, 0.989)
adj_bay_serop_20_35 <- epi.prev(nrow(sero[age_group == "[20,35)" &
test_result_cat=="Positive", ]),
nrow(sero[age_group == "[20,35)" & !is.na(test_result_cat) & outcome == "done", ]),
0.619, 0.989)
adj_bay_serop_5_20 <- epi.prev(nrow(sero[age_group == "[5,20)" &
test_result_cat=="Positive", ]),
nrow(sero[age_group == "[5,20)" & !is.na(test_result_cat) & outcome == "done", ]),
0.619, 0.989)
adj_bay_serop_0_5 <- epi.prev(nrow(sero[age_group == "[0,5)" & test_result_cat=="Positive",
]),
nrow(sero[age_group == "[0,5)" & !is.na(test_result_cat) & outcome == "done", ]),
0.619, 0.989)
```

BLCM Output

```
JAGS model summary statistics from 300000 samples (chains = 3; adapt+burnin = 11000):
Lower95 Median Upper95 Mean SD Mode MCerr MC%ofSD SSeff
se[1] 0.5682 0.61911 0.66909 0.61905 0.025775 -- 0.00015081 0.6 29209
sp[1] 0.98247 0.9894 0.99494 0.98908 0.0032768 -- 0.000018919 0.6 30000
se[2] 0.72266 0.77446 0.82388 0.77415 0.025912 -- 0.0001496 0.6 30000
sp[2] 0.9697 0.98578 0.99721 0.98457 0.0075763 -- 0.000043742 0.6 30000
AC.10 psrf
se[1] 0.0020527 0.99999
sp[1] -0.000081098 1.0001
se[2] 0.00016124 1.0002
sp[2] 0.00051855 1.0001
```

Examples of output are provided for the estimation of RDT sensitivity (Appendix Figures 5–8); estimation of RDT specificity (Appendix Figures 9–12); ELISA sensitivity (Appendix Figures 13–16), and ELISA specificity (Appendix Figures 17–20).

Discussion of Mortality Data

In the mortality survey we have tried to set a start of the recall period that would be easy to remember for the head of the households who would be the ones reporting about the deaths in their households. From previous experiences in mortality surveys, using special events or dates makes it easier for the respondents to provide more accurate information. Given that in the local calendar there was no other special event in January and February, we considered having January 1, 2019 as the starting date for the recall period. We acknowledge that there are some limitations with this approach and that recall bias could also affect the death rates specially for the prepandemic period, but we tried to tackle this through the training of the surveyors and the supervisors in the survey. For the selection of the starting of the pandemic period, and given the limited testing capacity available in country at the time, we considered that the virus was circulating earlier than March 13, the date of the first officially reported case, so we chose to select the start of month of March 2020 for the beginning of the Pandemic period. In regards of visual household-reported death excess starting in March 2020 (Figure), what we thought is that the first month of the pandemic the deaths were even more underreported (probably because of testing capacity and the delay on setting up the surveillance system for monitoring cases and deaths related to COVID-19). We suspect the virus was circulating and infecting humans before the first case was reported, which could explain the excess deaths in March 2020.

Finally, we considered determining an estimation of the infection fatality rate (IFR) using the results from the survey. Because we did not have reliable mortality data for Omdurman outside of the survey and could not be sure of the cause of death for the excess deaths estimated, we agreed that including this estimation could have been misleading. The IFR projected for Africa ranged between 0.004% in Botswana and Central African Republic to 0.67% in South Africa or 1.53% in Nigeria (*25*,*26*).

References

1. Report 39 - Characterising COVID-19 epidemic dynamics and mortality under-ascertainment in Khartoum, Sudan. Imperial College London. 2021 [cited 2021 Feb 2]. http://www.imperial.ac.uk/medicine/departments/school-public-health/infectious-disease-epidemiology/mrc-global-infectious-disease-analysis/covid-19/report-39-sudan/

2. World Health Organization. Population-based age-stratified seroepidemiological investigation protocol for coronavirus 2019 (COVID-19) infection. 2020 [cited 2021 Feb 2]. https://www.who.int/publications-detail-redirect/WHO-2019-nCoV-Seroepidemiology-2020.2

3. World population prospects - population division - United Nations. 2021 [cited 2021 Feb 2]. https://population.un.org/wpp/

4. Sudan FETP conducts targeted testing for COVID-19 in Khartoum State | TEPHINET. 2020 [cited 2021 Feb 2]. https://www.tephinet.org/sudan-fetp-conducts-targeted-testing-for-covid-19-in-khartoum-state

5. Kwenti TE, Njunda LA, Tsamul B, Nsagha SD, Assob NJ, Tufon KA, et al. Comparative evaluation of a rapid diagnostic test, an antibody ELISA, and a pLDH ELISA in detecting asymptomatic malaria parasitaemia in blood donors in Buea, Cameroon. Infect Dis Poverty. 2017;6:103. PubMed https://doi.org/10.1186/s40249-017-0314-2

6. Hajian-Tilaki K. Sample size estimation in diagnostic test studies of biomedical informatics. J Biomed Inform. 2014;48:193–204. PubMed https://doi.org/10.1016/j.jbi.2014.02.013

7. Higgins RL, Rawlings SA, Case J, Lee FY, Chan CW, Barrick B, et al. Longitudinal SARS-CoV-2 antibody study using the Easy Check COVID-19 IgM/IgG™ lateral flow assay. PLoS One. 2021;16:e0247797. PubMed https://doi.org/10.1371/journal.pone.0247797

8. Pavlova IP, Nair SS, Kyprianou N, Tewari AK. The rapid coronavirus antibody test: can we improve accuracy? Front Med (Lausanne). 2020;7:569. PubMed https://doi.org/10.3389/fmed.2020.00569

9. Cheung A, Dufour S, Jones G, Kostoulas P, Stevenson MA, Singanallur NB, et al. Bayesian latent class analysis when the reference test is imperfect. Rev Sci Tech. 2021;40:271–86. PubMed https://doi.org/10.20506/rst.40.1.3224

10. Hartnack S, Eusebi P, Kostoulas P. Bayesian latent class models to estimate diagnostic test accuracies of COVID-19 tests. J Med Virol. 2021;93:639–40. PubMed https://doi.org/10.1002/jmv.26405

11. Wang C, Lin X, Nelson KP. Bayesian hierarchical latent class models for estimating diagnostic accuracy. Stat Methods Med Res. 2020;29:1112–28. PubMed https://doi.org/10.1177/0962280219852649

12. Drewe JA, Tomlinson AJ, Walker NJ, Delahay RJ. Diagnostic accuracy and optimal use of three tests for tuberculosis in live badgers. PLoS One. 2010;5:e11196. PubMed https://doi.org/10.1371/journal.pone.0011196

13. Shim SR, Kim S-J, Lee J. Diagnostic test accuracy: application and practice using R software. Epidemiol Health. 2019;41:e2019007. PubMed https://doi.org/10.4178/epih.e2019007

14. FIND. (the global alliance for diagnostics). Clinical performance estimates for the 35 RDTs. 2020 [cited 2021 Aug 3]. https://www.finddx.org/wp-content/uploads/2021/04/COVID-Ab-DxData_2021-04-30.xlsx

15. US Federal Drug Administration. Serology test evaluation report for "STANDARD QCOVID-19 IgM/IgG Duo" from SD BIOSENSOR, Inc. 2020. 2020 [cited 2021 Aug 4]. https://www.accessdata.fda.gov/cdrh_docs/presentations/maf/maf3274-a001.pdf

16. Krüttgen A, Cornelissen CG, Dreher M, Hornef M, Imöhl M, Kleines M. Comparison of four new commercial serologic assays for determination of SARS-CoV-2 IgG. J Clin Virol. 2020;128:104394. PubMed https://doi.org/10.1016/j.jcv.2020.104394

17. Kohmer N, Westhaus S, Rühl C, Ciesek S, Rabenau HF. Brief clinical evaluation of six high-throughput SARS-CoV-2 IgG antibody assays. J Clin Virol. 2020;129:104480. PubMed https://doi.org/10.1016/j.jcv.2020.104480

18. US Federal Drug Administration. Serology test evaluation report for "SARS-COV-2 ELISA(IgG)" from Euroimmun. 2020 [cited 2021 Aug 4]. https://www.accessdata.fda.gov/cdrh_docs/presentations/maf/maf3246-a001.pdf

19. FIND. (the global alliance for diagnostics). Results for the evaluation of 16 Antibody ELISAs: overall sensitivity and specificity estimates. 2020 [cited 2021 Aug 3]. https://www.finddx.org/wp-content/uploads/2021/04/forestplot_All_ELISAS.pdf

20. Hartnack S, Eusebi P, Kostoulas P. Bayesian latent class models to estimate diagnostic test accuracies of COVID-19 tests. J Med Virol. 2021;93:639–40. PubMed https://doi.org/10.1002/jmv.26405

21. Cheung A, Dufour S, Jones G, Kostoulas P, Stevenson MA, Singanallur NB, et al. Bayesian latent class analysis when the reference test is imperfect. Rev Sci Tech. 2021;40:271–86. PubMed https://doi.org/10.20506/rst.40.1.3224

22. Wang C, Lin X, Nelson KP. Bayesian hierarchical latent class models for estimating diagnostic accuracy. Stat Methods Med Res. 2020;29:1112–28. PubMed https://doi.org/10.1177/0962280219852649

23. Denwood MJ. runjags: An R package providing interface utilities, model templates, parallel computing methods and additional distributions for MCMC Models in JAGS. J Stat Softw. 2016;71:1–25. https://doi.org/10.18637/jss.v071.i09

24. Johnson W, Su C-L. Betabuster. 2020 [cited 2021 Aug 4]. https://cadms.vetmed.ucdavis.edu/sites/g/files/dgvnsk5071/files/inline-files/betabuster012006.zip.

25. Mutevedzi PC, Kawonga M, Kwatra G, Moultrie A, Baillie V, Mabena N, et al. Estimated SARS-CoV-2 infection rate and fatality risk in Gauteng Province, South Africa: a population-based seroepidemiological survey. Int J Epidemiol. 2021;dyab217; Epub ahead of print. PubMed https://doi.org/10.1093/ije/dyab217

26. Onovo AA, Kalaiwo A, Obanubi C, Odezugo G, Estill J, Keiser O. Estimates of the COVID-19 infection fatality rate for 48 African countries: a model-based analysis. BioMed. 2021;1:63–79. https://doi.org/10.3390/biomed1010005

Appendix Table 1. Causes of death by age group in study of mortality and SARS-CoV-2 seroprevalence, Sudan*

Cause of death	<20 y	20–34 y	35–49 y	≥50 y	Total
Accident / Trauma	4 (12.9)	7 (22.6)	6 (19.4)	14 (45.2)	31 (100.0)
Cancer	1 (4.3)	2 (8.7)	5 (21.7)	15 (65.2)	23 (100.0)
Chronic kidney disease	0 (0.0)	0 (0.0%)	0 (0.0)	1 (100.0)	1 (100.0)
COVID-19	1 (11.1)	0 (0.0)	1 (11.1)	7 (77.8)	9 (100.0)
Diarrhea	1 (14.3)	1 (14.3)	0 (0.0)	5 (71.4)	7 (100.0)
Don't know	7 (13.0)	3 (5.6)	4 (47.4)	40 (74.1)	54 (100.0)
Isolated fever / Malaria	1 (7.7)	2 (15.4)	2 (15.4)	8 (61.5)	13 (100.0)
Malnutrition	1 (50.0)	0 (0.0)	0 (0.0)	1 (50.0)	2 (100.0)
NCD	1 (1.2)	2 (2.3)	12 (14.0)	71 (82.6)	86 (100.0)
Neonatal death/disease	11 (91.7)	1 (8.3)	0 (0.0)	0 (0.0)	12 (100.0)
Others	6 (11.5)	6 (11.5)	7 (13.5)	33 (63.5)	52 (100.0)
Pregnancy/childbirth related deaths	3 (50.0)	2 (33.3)	1 (16.7)	0 (0.0)	6 (100.0)
Respiratory diseases	5 (29.4)	1 (5.9)	2 (11.8)	9 (52.9)	17 (100.0)
Unknown	9 (0.0)	0 (0.0)	0 (0.0)	1 (100.0)	1 (100.0)
Violence	1 (20)	3 (60.0)	0 (0.0)	1 (20.0)	5 (100.0)
Total	43 (13.5)	30 (9.4)	40 (12.5)	206 (64.6)	319 (100.0)

*Values are expressed as no. (%). COVID-19, coronavirus disease; NCD, non-communicable disease; SARS-CoV-2, severe acute respiratory syndrome coronavirus 2.

Appendix Table 2. Causes of death by age group and study period in study of mortality and SARS-CoV-2 seroprevalence, Sudan*

	<20 y		20–34 y		35–49 y		≥50 y		Total	
Cause of death	PREP	PAND	PREP	PAND	PREP	PAND	PREP	PAND	PREP	PAND
Accident / trauma	1 (5)	3 (13)	1 (10)	6 (30)	5 (31)	1 (4)	6 (8)	8 (6)	13 (10)	18 (9)
Cancer	1 (5)	0 (0)	1 (10)	1 (5)	2 (12)	3 (12)	6 (8)	9 (7)	10 (8)	13 (7)
Chronic kidney disease	0 (0)	0 (0)	0 (0)	0 (0)	0 (0)	0 (0)	1 (1)	0 (0)	1 (1)	0 (0)
COVID-19	0 (0)	1 (4)	0 (0)	0 (0)	0 (0)	1 (4)	0 (0)	7 (6)	0 (0)	9 (5)
Diarrhea	0 (0)	1 (4)	1 (10)	0 (0)	0 (0)	0 (0)	1 (1)	4 (3)	2 (2)	5 (3)
Don't know	2 (10)	5 (22)	1 (10)	2 (10)	2 (12)	2 (8)	19 (24)	21 (17)	24 (19)	30 (16)
Isolated fever / malaria	1 (5)	0 (0)	1 (10)	1 (5)	1 (6)	1 (4)	2 (2)	6 (5)	5 (4)	8 (4)
Malnutrition	1 (5)	0 (0)	0 (0)	0 (0)	0 (0)	0 (0)	0 (0)	1 (1)	1 (1)	1 (1)
NCD	0 (0)	1 (4)	0 (0)	2 (10)	5 (31)	7 (29)	28 (35)	43 (34)	33 (26)	53 (27)
Neonatal death/disease	6 (30)	5 (22)	0 (0)	1 (5)	0 (0)	0 (0)	0 (0)	0 (0)	6 (5)	6 (3)
Others	2 (10)	4 (17)	2 (20)	4 (20)	1 (6)	6 (25)	15 (19)	18 (14)	20 (16)	32 (17)
Pregnancy/childbirth related	2 (10)	1 (4)	2 (20)	0 (0)	0 (0)	1 (4)	0 (0)	0 (0)	4 (3)	2 (1)
Respiratory infections	4 (20)	1 (4)	0 (0)	1 (5)	0 (0)	2 (8)	2 (2)	7 (6)	6 (5)	11 (6)
Unknown	0 (0)	0 (0)	0 (0)	0 (0)	0 (0)	0 (0)	0 (0)	1 (1)	0 (0)	1 (1)
Violence	0 (0)	1 (4)	1 (10)	2 (10)	0 (0)	0 (0)	0 (0)	1 (1)	1 (1)	4 (2)
Total (n)	20 (100)	23 (100)	10 (100)	20 (100)	16 (100)	24 (100)	80 (100)	126 (100)	126 (100)	193 (100)

*No major differences were found between the two periods stratified by age groups. PREP, Prepandemic period, January 1, 2019– February 29, 2020. PAND, pandemic period, March 1, 2020, until the end of the survey. NCD, non-communicable disease.

The most common cause of death as reported by the heads of households were non–communicable diseases, followed by trauma/accidents and cancer

Appendix Table 3. Summary of symptoms reported before death in prepandemic and pandemic periods in study of mortality and SARS-CoV-2 seroprevalence, Sudan*

Symptom	Prepandemic, n (%)	Pandemic, n (%)	p value
Fever	16 (12.7)	32 (16.6)	<0.001
Cough	7 (5.6)	18 (9.3)	0.288
Shortness of breath	19 (15.1)	28 (14.5)	0.873
Weakness/Fatigue	14 (11.1)	30 (15.5)	0.32
Extreme fatigue	6 (4.8)	22 (11.4)	0.044
Headaches	8 (6.3)	20 (10.4)	0.233
Muscle pain	6 (4.8)	23 (11.9)	0.044
Sore throat	3 (2.4)	3 (1.6)	0.684
Runny nose	1 (0.8)	1 (0.5)	>0.999
Loss appetite	6 (4.8)	20 (10.4)	0.094
Diarrhea	7 (5.6)	11 (5.7)	>0.999
Change mental state	5 (4.0)	5 (2.6)	0.524
Loss taste/odour	1 (0.8)	1 (0.5)	>0.999
No symptoms	51 (40.5)	67 (4.7)	0.343
Don't know	25 (19.8)	40 (20.7)	0.888

*Reported symptoms before death were similar during both periods, apart from more commonly reported extreme fatigue and muscle pain during the pandemic period.

Appendix Table 4. Reported comorbidities for deaths during the prepandemic and pandemic periods in study of mortality and SARS-CoV-2 seroprevalence, Sudan*

Location	Prepandemic, n (%)	Pandemic, n (%)	p value
Asthma	0 (0.0)	3 (3.6)	0.553
Autoimmune disease (polyarthritis, Crohn's disease, lupus, multiple sclerosis…)	1 (2.1)	2 (2.4)	>0.999
Cancer	3 (6.2)	6 (7.1)	>0.999
Chronic lung disease	0 (0.0)	1 (1.2)	>0.999
Congestive heart failure	3 (6.2)	1 (1.2)	0.136
Coronary heart disease	3 (6.2)	7 (8.3)	0.747
Current smoker	0 (0.0)	1 (1.2)	>0.999
Diabetes	11 (22.9)	19 (22.6)	>0.999
Hepatitis B	0 (0.0)	1 (1.2)	>0.999
Hypertension	16 (33.3)	24 (28.6)	0.563
Kidney disease	5 (10.4)	(7 (8.3)	0.757
Other	6 (12.5)	12 (14.3)	>0.999

*We saw no differences between the 2 periods for rates of comorbidities.

Appendix Table 5. Access to healthcare in the prepandemic and pandemic periods in study of mortality and SARS-CoV-2 seroprevalence, Sudan*

Access to healthcare	Pre-pandemic, n (%)	Pandemic, n (%)	p value
Health center	4 (5.0)	10 (8.3)	0.413
Hospital	66 (82.5)	90 (75.0)	0.227
Other	1 (1.2)	1 (0.8)	>0.999
Pharmacist	0 (0.0)	1 (0.8)	>0.999
Self-medication: modern medicine	8 (10.0)	18 (15.0)	0.392
Self-medication: traditional medicine	1 (1.2)	0 (0.0)	0.400

*No major differences in the access to health facilities and place of death were seen between the two periods.

Appendix Table 6. Location of death for the prepandemic and pandemic periods in study of mortality and SARS-CoV-2 seroprevalence, Sudan*

Location of death	Prepandemic, n (%)	Pandemic, n (%)	p value
Don't know	1 (0.8)	0 (0.0)	0.395
Health center	1 (0.8)	3 (1.6)	>0.999
Home	55 (43.7)	91 (47.2)	0.567
Hospital	63 (50)	87 (45.1)	0.423
On the way to the health center/hospital	1 (0.8)	6 (3.1)	0.251
Other	5 (4)	6 (3.1)	0.758

*No major differences in the place of death were seen between the two periods.

Appendix Table 7. Status of consent by age group and sex to participate in study of SARS-CoV-2 seroprevalence, Sudan

	Consented, n (%)		Refused, n (%)		Absent, n (%)	
Age group	F	M	F	M	F	M
<5 y	130 (43.5)	169 (56.5)	66 (58.4)	47 (41.6)	14 (48.3)	15 (51.7)
5–19 y	424 (54.0)	361 (46.0)	123 (46.4)	142 (53.6)	124 (43.5)	161 (56.5)
20–34 y	383 (60.9)	246 (39.1)	74 (40.9)	107 (59.1)	59 (26.5)	164 (73.5)
35–49 y	217 (63.5)	125 (36.5)	20 (26.0)	57 (74.0)	27 (27.0)	73 (73.0)
≥50 y	159 (49.8)	160 (50.2)	37 (44.6)	46 (55.4)	19 (24.1)	60 (75.9)
Total	2,374 (62.3)		719 (18.9)		716 (18.8)	

Appendix Table 8. Risk factors associated with a positive SARS-Cov-2 rapid antibody test, Sudan*

		Adjusted OR	
Risk factor	Crude OR (95% CI)	OR (95% CI)	p-value
Age†	1.01 (1.01–1.02)	1.01 (1.00–1.02)	0.001
Sex‡	0.82 (0.66–1.02)	0.84 (0.67–1.05)	0.127
Past medical history§	1.30 (1.00–1.69)	1.06 (0.79–1.42)	0.703
Exposed to case in household¶	1.68 (1.35–2.08)	1.61 (1.21–2.01)	<0.001

*OR = odds ratio.
†Continuous variable.
‡Reference value is female.
§Reference value has a medical history.
¶Reference value is have been exposed to a case within the household.
Other than age, living with person who was seropositive led to a 1.68 (odds ratio [OR] 95% CI 1.35–2.08, p < 0.001 S7 Table) fold increase in the odds of being seropositive. Among all 555 included households, 364 (65.6%) had ≥1 positive household member and 203 household (36.6%) at least two. Sex was not a significant predictor for seroprevalence (p = 0.127).

Appendix Table 9. Summary of positive rapid serologic testing by antibody status in study of SARS-CoV-2 seroprevalence, Sudan*

Age group	Positive IgG ,n (%)	Positive IgG and IgM ,n (%)	Positive IgM ,n (%)	Total ,n (%)
<5 y	52 (92.9)	1 (1.8)	3 (5.4)	56 (100.0)
5–19 y	218 (90.8)	19 (7.9)	3 (1.2)	240 (100.0)
20–34 y	175 (78.5)	27 (12.1)	21 (9.4)	223 (100.0)
35–49 y	112 (83.0)	17 (12.6)	6 (4.4)	135 (100.0)
≥50 y	134 (83.8)	22 (13.8)	4 (2.5)	160 (100.0)
Total	691 (84.9)	86 (10.6)	37 (4.5)	814 (100.0)

*Among those who tested positive, most presented IgG antibodies (84.9%), whereas 10.6% tested positive for both antibodies and 4.5% for IgM only.

Appendix Table 10. Summary of ELISA and rapid test results in study of SARS-CoV-2 seroprevalence, Sudan*

Result	ELISA negative, n (%)	ELISA positive, n (%)	Total, n (%)
RDT negative	443 (76.2)	138 (23.8)	581 (100.0)
RDT positive	60 (24.6)	184 (75.4)	244 (100.0)
Total	503 (61.0)	322 (39.0)	825 (100.0)

* ELISA, enzyme-linked immunosorbent assay. RDT, rapid diagnostic test.
825 samples were tested with the ELISA, of which 244 (29.6%) and 322 (39.0%) were positive for SARS-CoV-2 IgG by the RDT and ELISA respectively). Among 198 cases with discordant results considering ELISA as standard, 60 (30.0%) were false positives and 138 (70.0%) false negatives according to the RDT. (serology).

Appendix Table 11. Prevalence of SARS-CoV-2 antibodies based on rapid serologic test by age group and sex in study of SARS-CoV-2 seroprevalence, Sudan*

Age group	Prevalence in female patients, % (95% CI)	Prevalence in male patients, % (95% CI)	Total, % (95% CI)
<5 y	22.9 (17.0–30.1)	15.2 (9.7–23.1)	18.6 (14.0–24.2)
5–19 y	32.1 (27.0–37.6)	28.7 (23.3–34.8)	30.5 (26.3–35.1)
20–34 y	32.0 (27.3–37.1)	40.6 (33.0–48.7)	35.3 (30.7–40.3)
35–49 y	40.5 (34.5–46.9)	37.4 (28.6–47.1)	39.4 (34.9–44.1)
≥50 y	50.4 (41.5–59.2)	50.1 (42.8–57.4)	50.2 (44.0–56.5)

```
        proportion        95%-CI
Study 1      0.667 [0.563; 0.760]
Study 2      0.717 [0.642; 0.784]
Study 3      0.577 [0.521; 0.632]
Study 4      0.820 [0.787; 0.851]
Study 5      0.805 [0.752; 0.851]
Study 6      0.896 [0.866; 0.922]
Study 7      0.767 [0.577; 0.901]

Number of studies combined: k = 7

                     proportion         95%-CI
Random effects model      0.766 [0.678; 0.836]

Quantifying heterogeneity:
 tau^2 = 0.3118; tau = 0.5584; I^2 = 95.0% [91.9%; 96.9%]; H = 4.48 [3.52; 5.70]

Test of heterogeneity:
      Q d.f.  p-value             Test
 120.58    6 < 0.0001        Wald-type
 126.95    6 < 0.0001 Likelihood-Ratio

Details on meta-analytical method:
- Random intercept logistic regression model
- Maximum-likelihood estimator for tau^2
- Logit transformation
- Clopper-Pearson confidence interval for individual studies
```

Appendix Figure 1. Output of the metaprop function for the estimation of sensitivity of rapid diagnostic test in study of SARS-CoV-2 seroprevalence and patient deaths, Sudan.

```
         proportion         95%-CI
Study 1       1.000 [0.964; 1.000]
Study 2       0.990 [0.964; 0.999]
Study 3       0.976 [0.931; 0.995]
Study 4       0.991 [0.976; 0.997]
Study 5       0.997 [0.981; 1.000]
Study 6       0.984 [0.964; 0.995]
Study 7       0.988 [0.932; 1.000]

Number of studies combined: k = 7

                     proportion         95%-CI
Random effects model      0.990 [0.983; 0.994]

Quantifying heterogeneity:
 tau^2 = 0; tau = 0; I^2 = 0.0% [0.0%; 52.4%]; H = 1.00 [1.00; 1.45]

Test of heterogeneity:
    Q d.f. p-value             Test
 3.68    6  0.7198        Wald-type
 6.52    6  0.3675 Likelihood-Ratio
```

Appendix Figure 2. Output of the metaprop function for the estimation of the specificity of rapid diagnostic test in study of SARS-CoV-2 seroprevalence and patient deaths, Sudan.

```
        proportion         95%-CI
Study 1      0.864 [0.651; 0.971]
Study2       0.900 [0.735; 0.979]
Study 3      0.711 [0.557; 0.836]
Study 4      0.598 [0.490; 0.699]

Number of studies combined: k = 4

                     proportion         95%-CI
Random effects model      0.769 [0.611; 0.876]

Quantifying heterogeneity:
 tau^2 = 0.3653; tau = 0.6044; I^2 = 74.4% [28.8%; 90.8%]; H = 1.98 [1.19; 3.30]

Test of heterogeneity:
     Q d.f. p-value             Test
 11.74    3  0.0083        Wald-type
 14.58    3  0.0022 Likelihood-Ratio
```

Appendix Figure 3. Output of the metaprop function for the estimation of the sensitivity for the ELISA in study of SARS-CoV-2 seroprevalence and patient deaths, Sudan.

```
       proportion         95%-CI
Study 1      0.962 [0.870; 0.995]
Study2       1.000 [0.955; 1.000]
Study 3      1.000 [0.846; 1.000]
Study 4      0.993 [0.976; 0.999]

Number of studies combined: k = 4

                     proportion         95%-CI
Random effects model      0.991 [0.977; 0.997]

Quantifying heterogeneity:
 tau^2 = 0; tau = 0; I^2 = 0.0% [0.0%; 84.7%]; H = 1.00 [1.00; 2.56]

Test of heterogeneity:
    Q d.f. p-value             Test
 3.00    3  0.3917        Wald-type
 4.76    3  0.1904 Likelihood-Ratio
```

Appendix Figure 4: Output of the metaprop function for the estimation of the specificity of the ELISA in study of SARS-CoV-2 seroprevalence and patient deaths, Sudan.

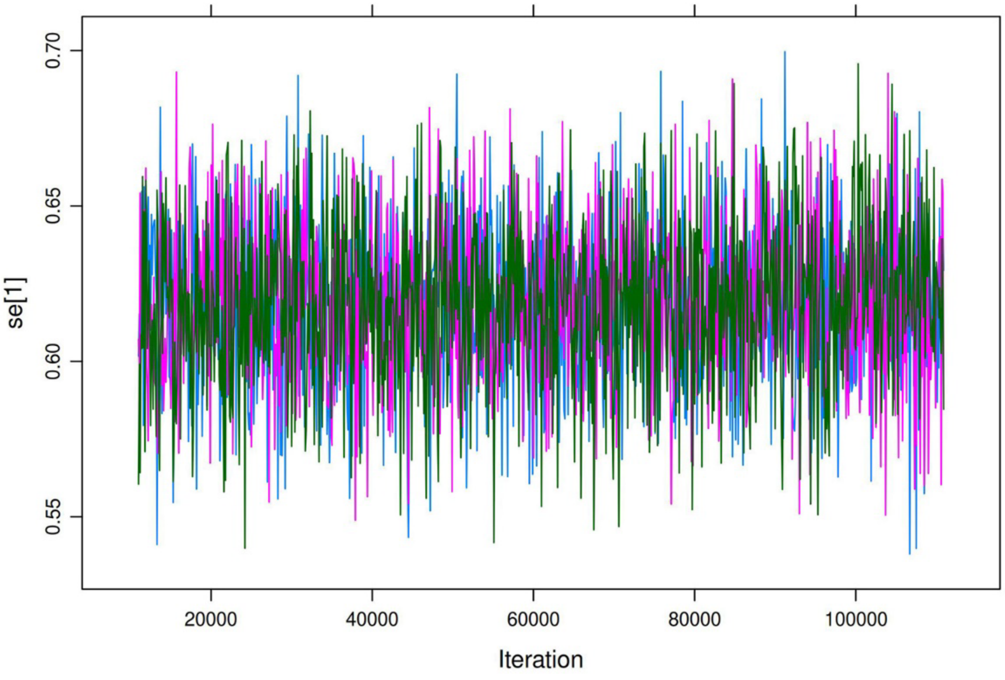

Appendix Figure 5: Output of the runjags function as a trace plot of the 3 chains to estimate the sensitivity of the rapid diagnostic test in study of SARS-CoV-2 seroprevalence and patient deaths, Sudan.

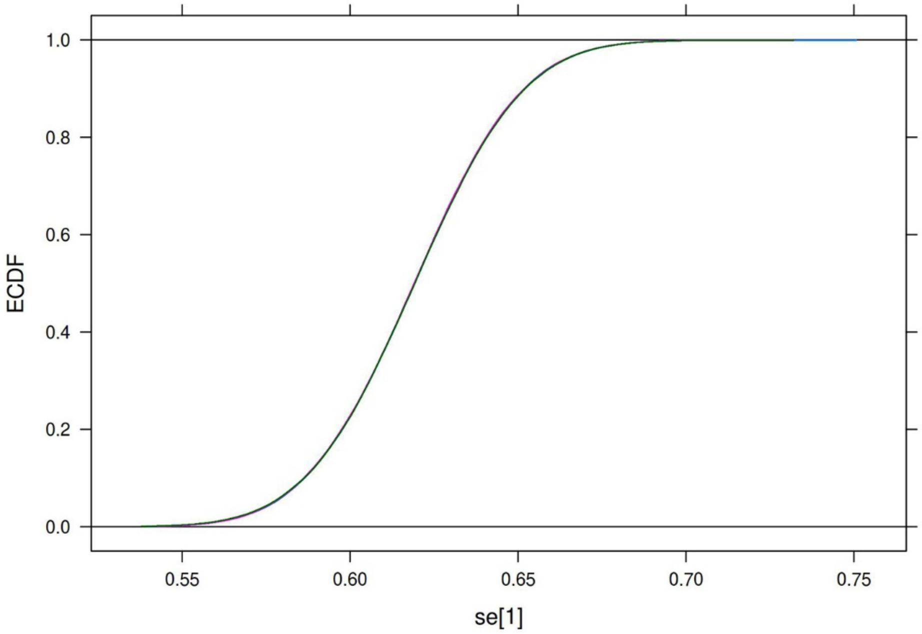

Appendix Figure 6: Output of the runjags function as an ECDF plot showing the 3 overlapping chains to estimate the sensitivity of the rapid diagnostic test in study of SARS-CoV-2 seroprevalence and patient deaths, Sudan.

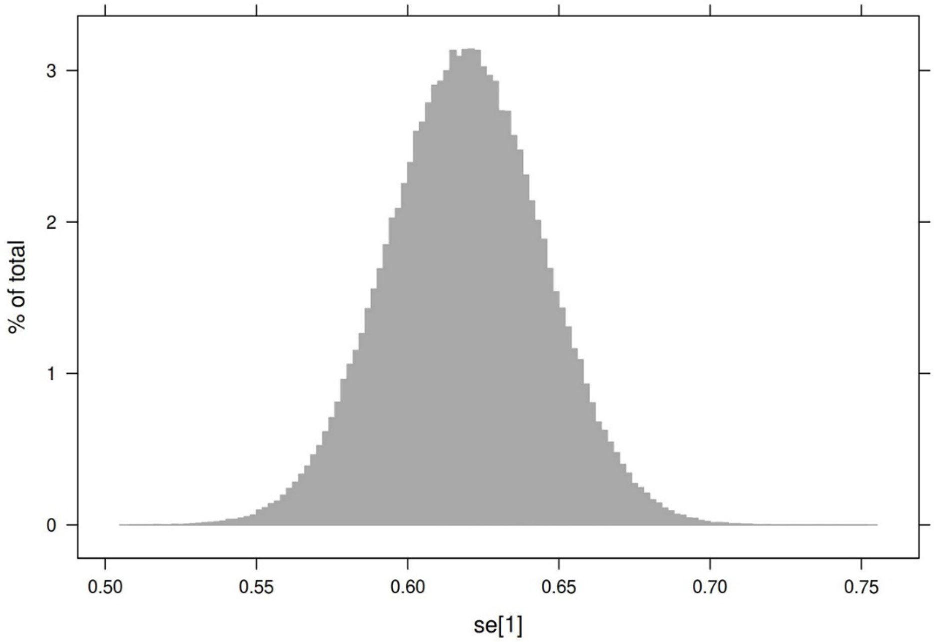

Appendix Figure 7: Output of the runjags function as histograms of the combined chains to estimate the sensitivity of rapid diagnostic test in study of SARS-CoV-2 seroprevalence and patient deaths, Sudan.

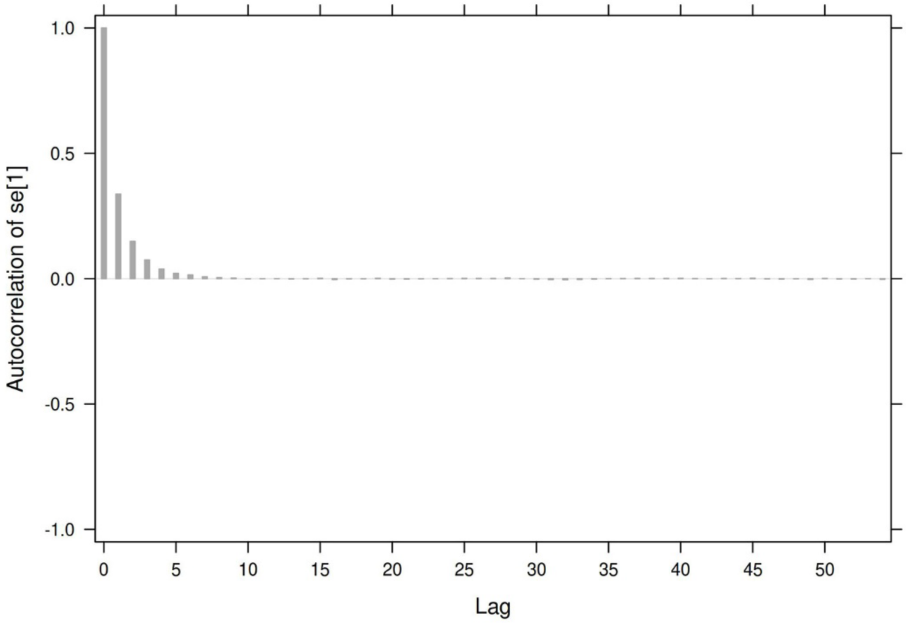

Appendix Figure 8: Output of the runjags function as an autocorrelation plot to estimate the sensitivity of rapid diagnostic test in study of SARS-CoV-2 seroprevalence and patient deaths, Sudan.

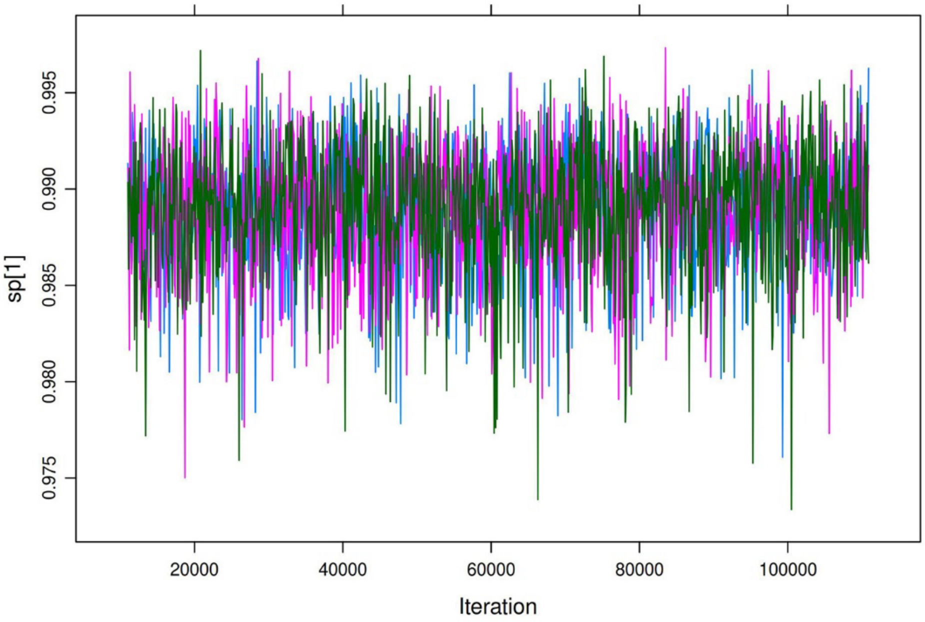

Appendix Figure 9: Output of the runjags function as a trace plot of 3 stationary chains to estimate the specificity of rapid diagnostic test in study of SARS-CoV-2 seroprevalence and patient deaths, Sudan.

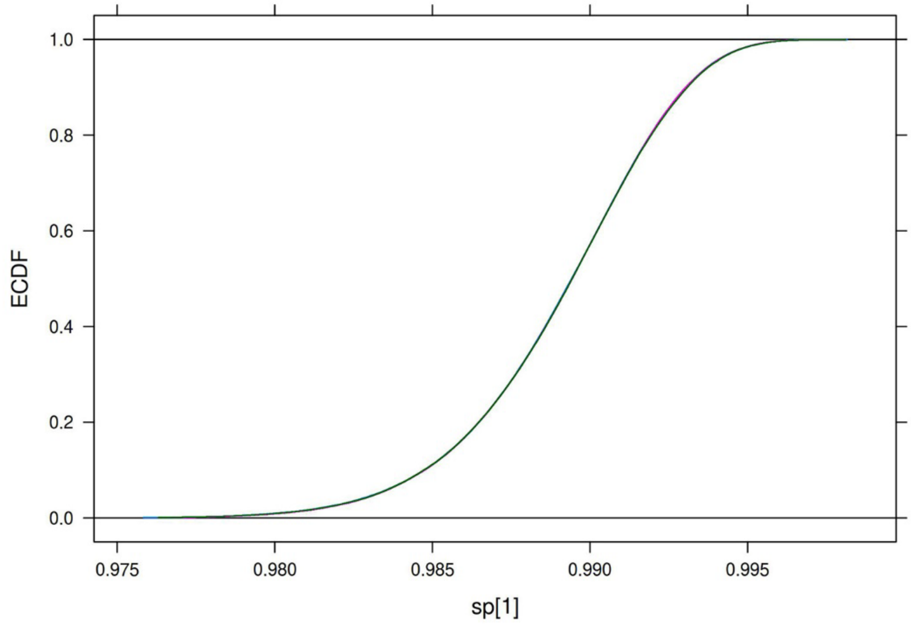

Appendix Figure 10: Output of the runjags function as an ECDF plot showing the 3 overlapping chains to estimate the specificity of rapid diagnostic test in study of SARS-CoV-2 seroprevalence and patient deaths, Sudan.

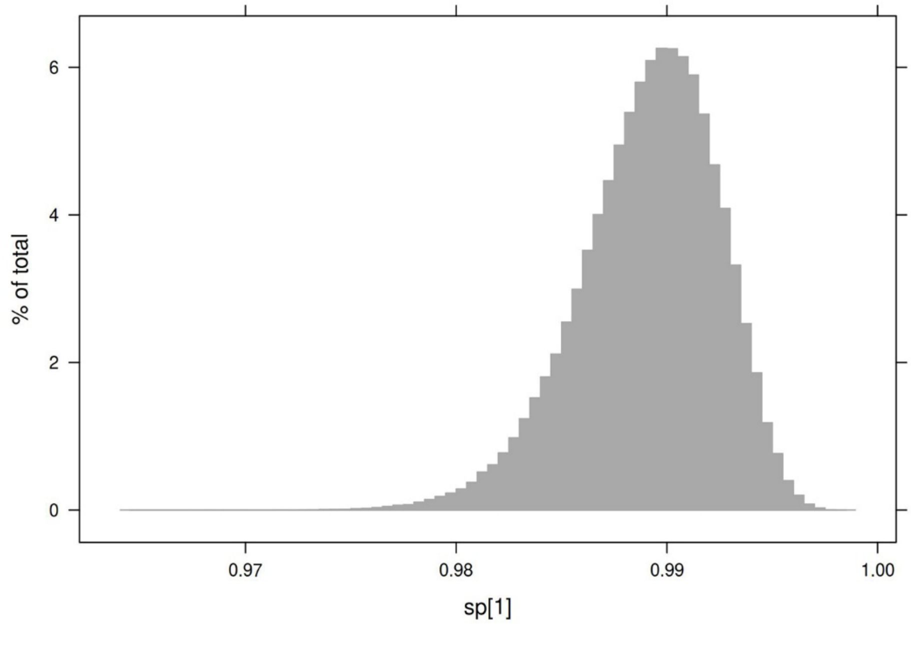

Appendix Figure 11: Output of the runjags function as histograms of the combined chains to estimate the specificity of rapid diagnostic test in study of SARS-CoV-2 seroprevalence and patient deaths, Sudan.

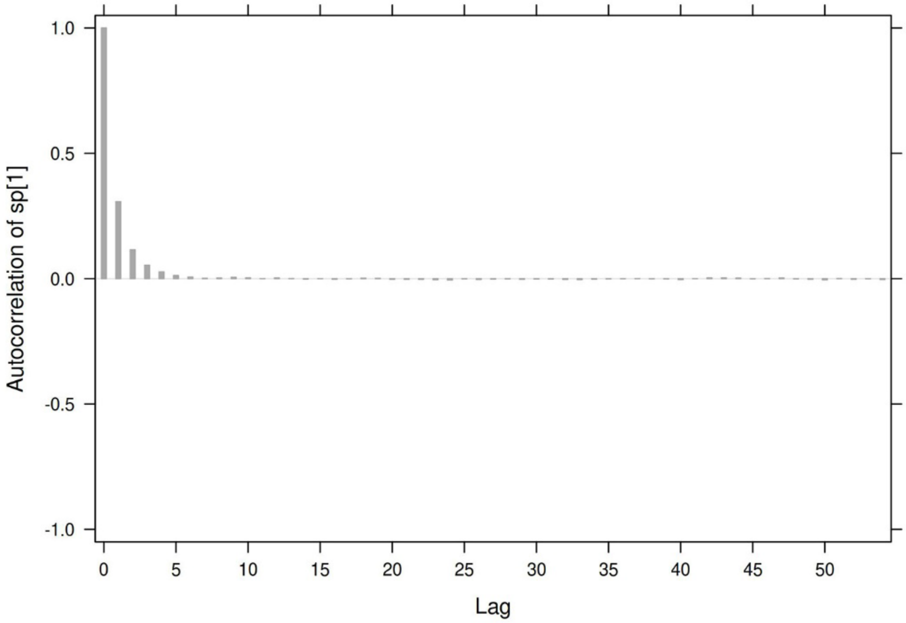

Appendix Figure 12: Output of the runjags function as an autocorrelation plot to estimate the specificity of rapid diagnostic test in study of SARS-CoV-2 seroprevalence and patient deaths, Sudan.

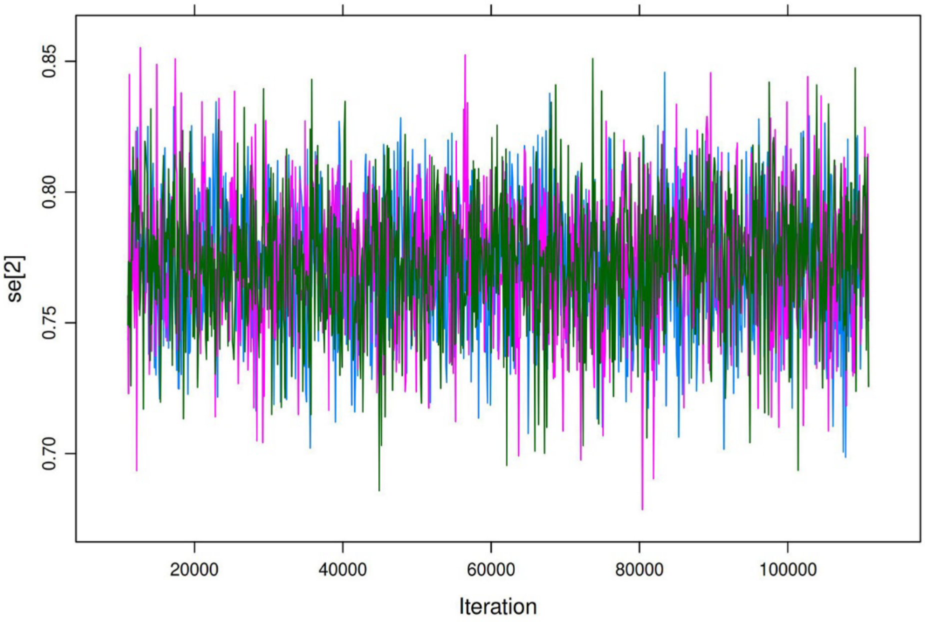

Appendix Figure 13: Output of the runjags function as a trace plot of the 3 stationary chains to estimate the sensitivity of the ELISA in study of SARS-CoV-2 seroprevalence and patient deaths, Sudan.

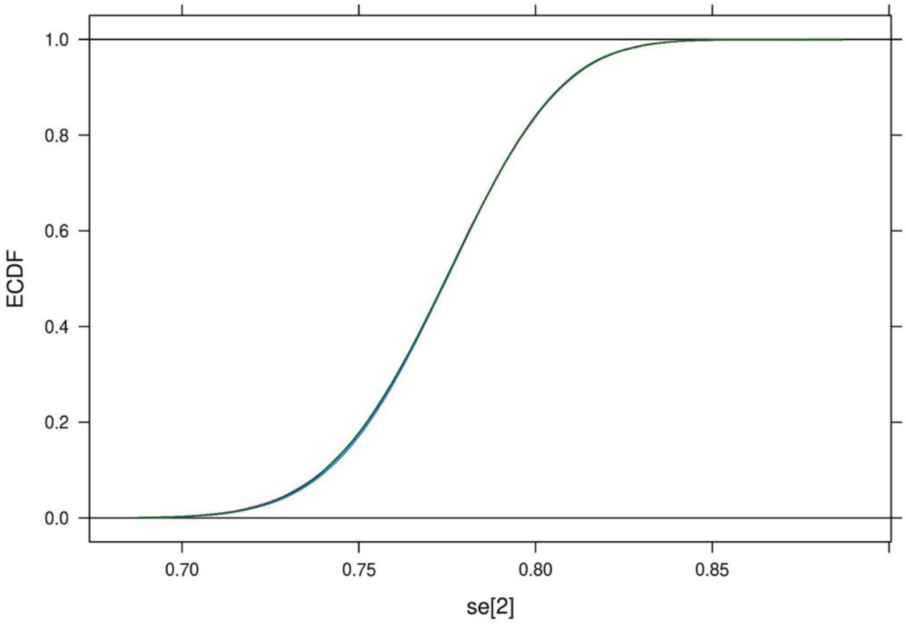

Appendix Figure 14: Output of the runjags function as an ECDF plot showing the 3 overlapping chains to estimate the sensitivity of the ELISA in study of SARS-CoV-2 seroprevalence and patient deaths, Sudan.

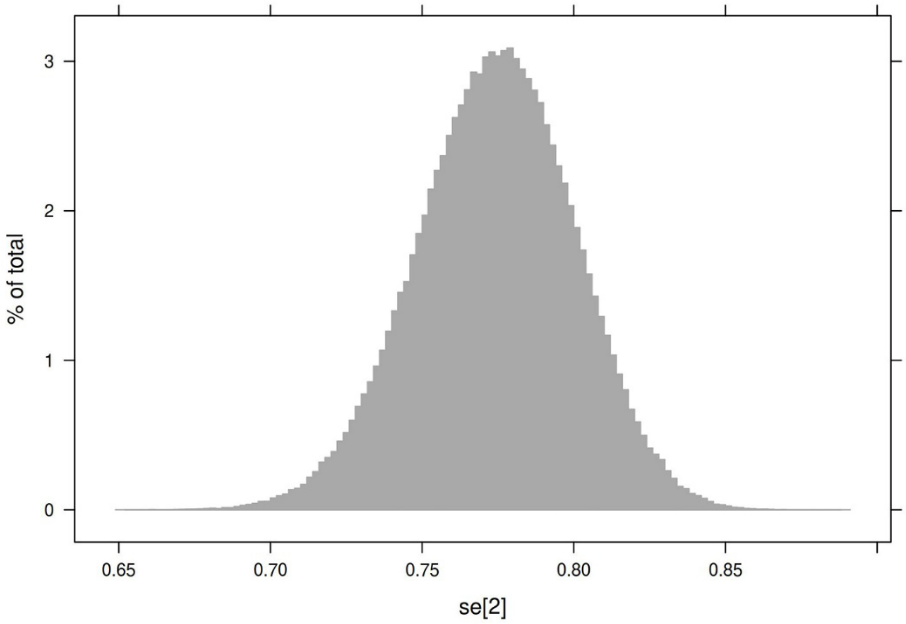

Appendix Figure 15: Output of the runjags function as histograms of the combined chains to estimate the sensitivity of the ELISA in study of SARS-CoV-2 seroprevalence and patient deaths, Sudan..

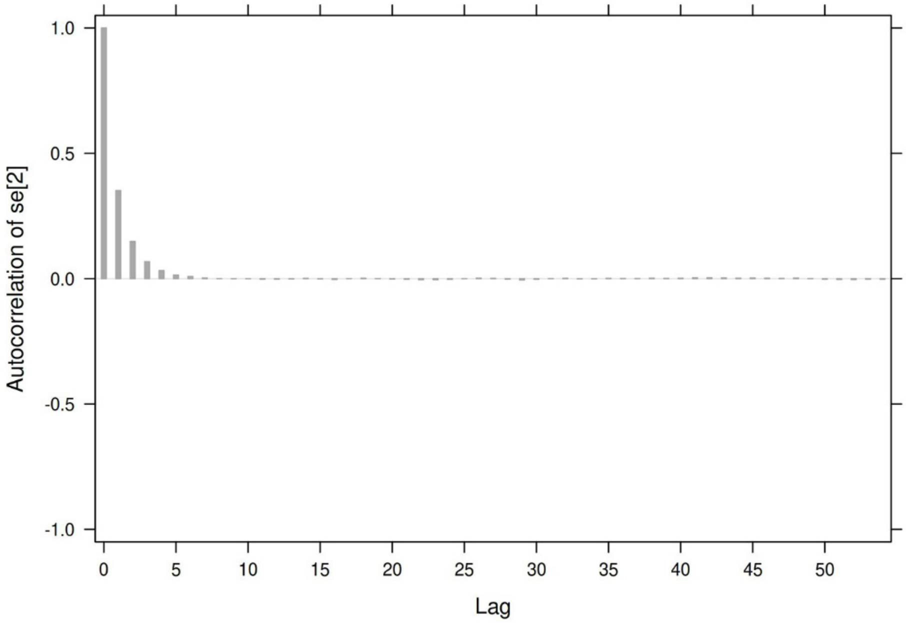

Appendix Figure 16: Output of the runjags function as an autocorrelation plot to estimate the sensitivity of the ELISA in study of SARS-CoV-2 seroprevalence and patient deaths, Sudan.

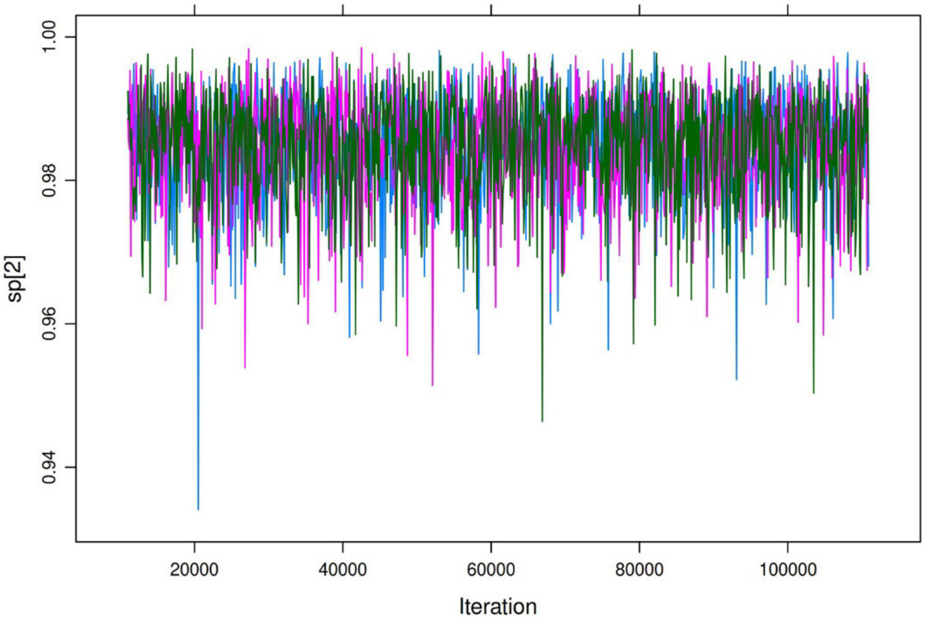

Appendix Figure 17: Output of the runjags function as a trace plot of the 3 stationary chains to estimate the specificity of the ELISA in study of SARS-CoV-2 seroprevalence and patient deaths, Sudan.

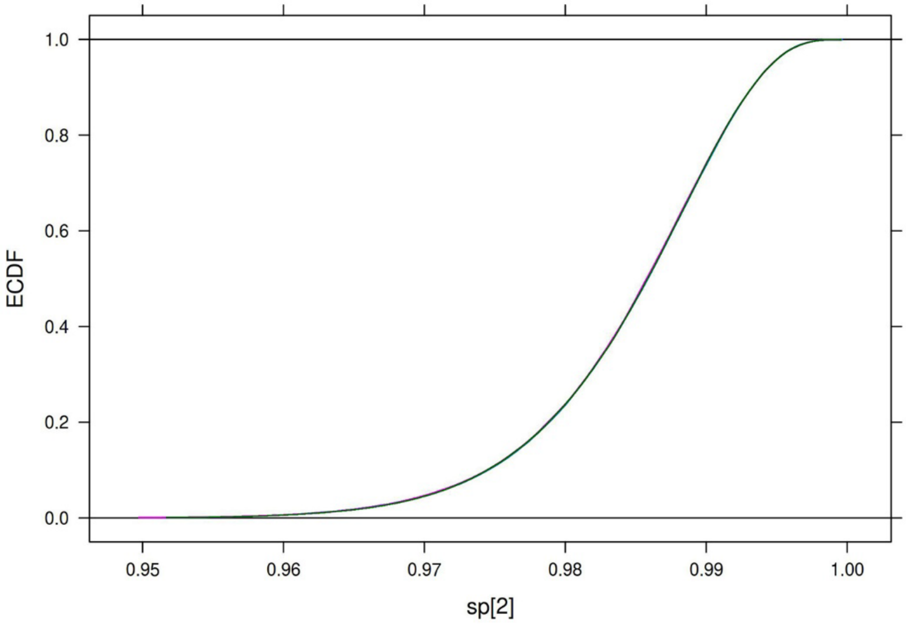

Appendix Figure 18: Output of the runjags function as an ECDF plot showing the 3 overlapping chains to estimate the specificity of the ELISA in study of SARS-CoV-2 seroprevalence and patient deaths, Sudan.

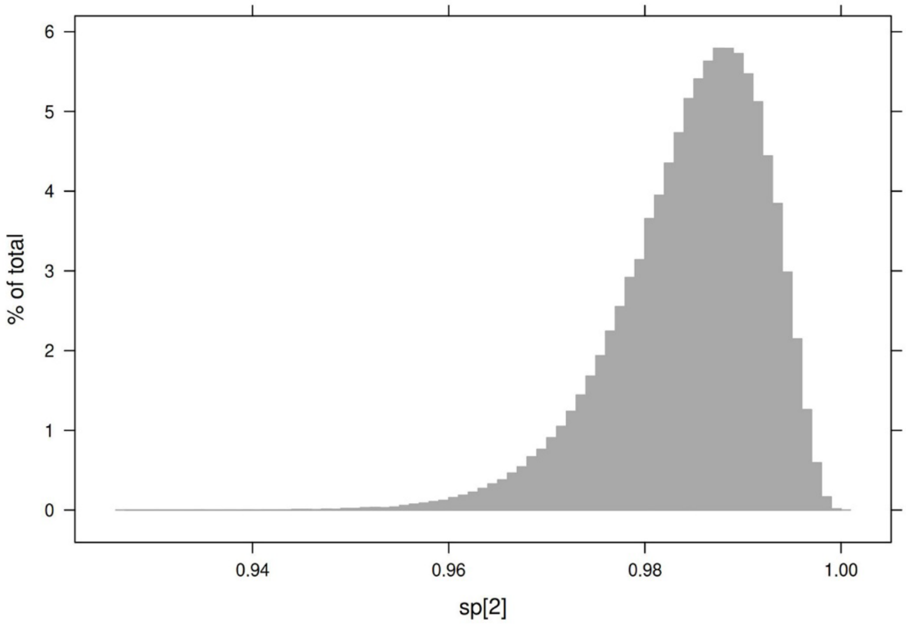

Appendix Figure 19: Output of the runjags function as histograms of the combined chains to estimate the specificity of the ELISA in study of SARS-CoV-2 seroprevalence and patient deaths, Sudan.

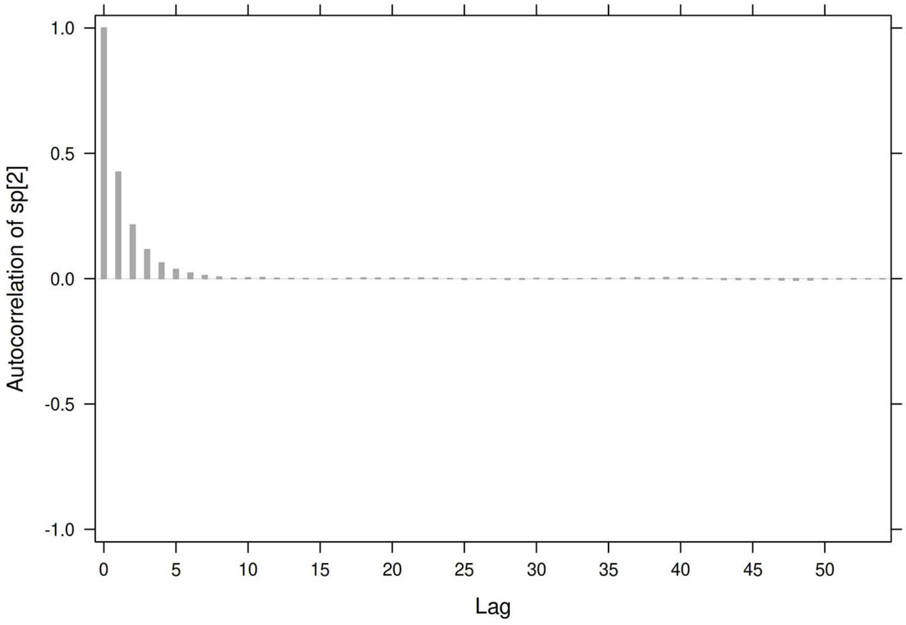

Appendix Figure 20: Output of the runjags function as an autocorrelation plot to estimate the specificity of the ELISA in study of SARS-CoV-2 seroprevalence and patient deaths, Sudan.

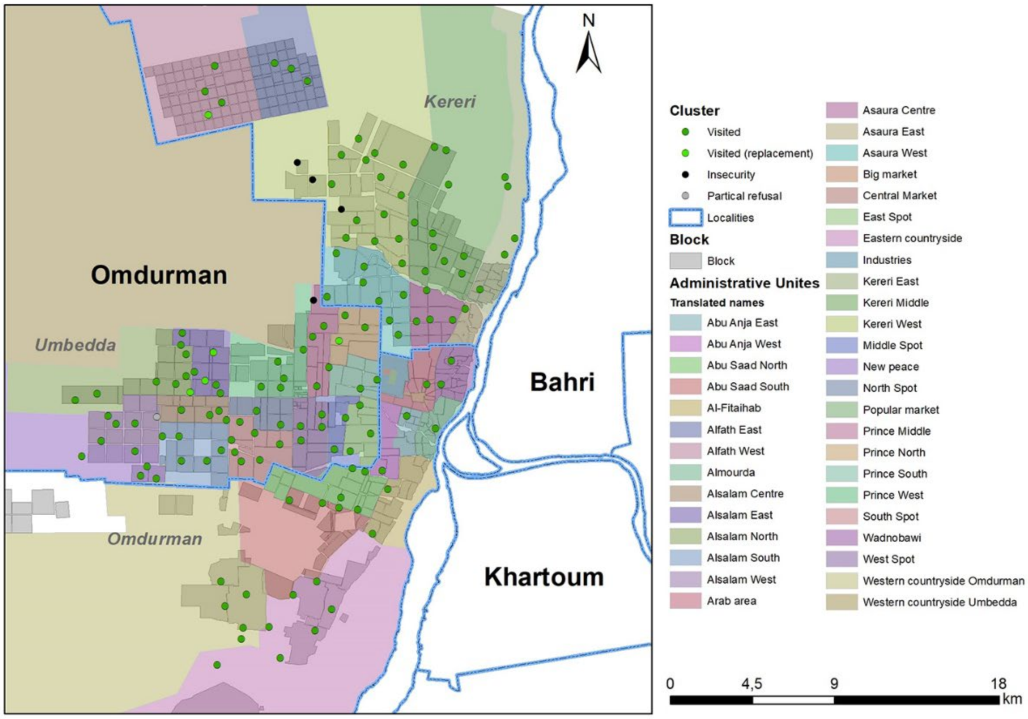

Appendix Figure 21. Distribution of clusters of SARS-CoV-2 seroprevalence in the 34 administrative units in greater Omdurman, Sudan.

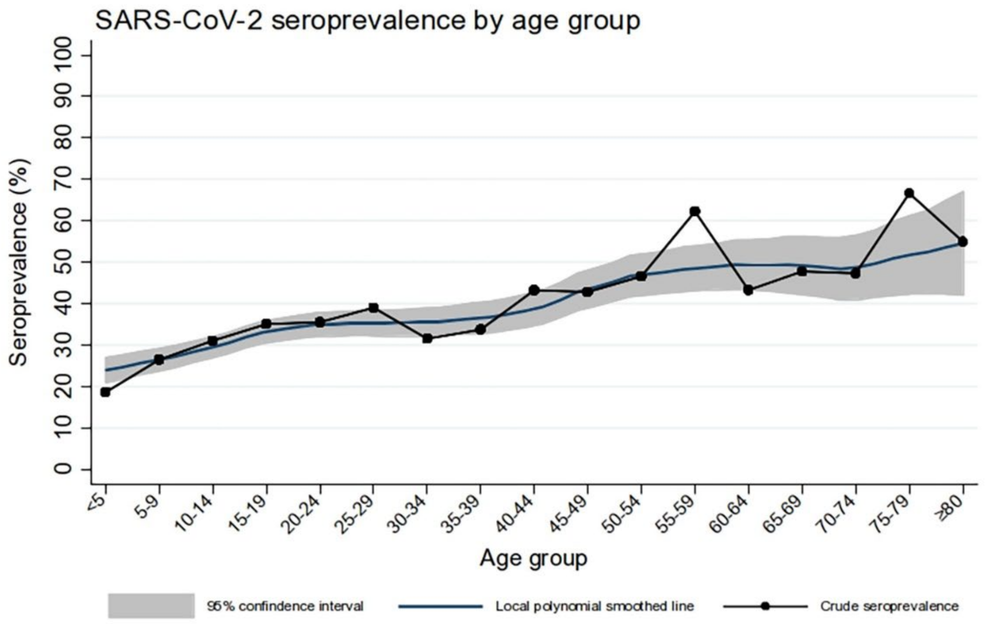

Appendix Figure 22. SARS-CoV-2 seroprevalence by age group in study of SARS-CoV-2 seroprevalence and patient deaths, Sudan.
